# Hierarchical determinants in cytotoxic necrotizing factor (CNF) toxins driving Rho G-protein deamidation versus transglutamination

**DOI:** 10.1128/mbio.01221-24

**Published:** 2024-06-26

**Authors:** Nicholas B. Handy, Yiting Xu, Damee Moon, Jacob J. Sowizral, Eric Moon, Mengfei Ho, Brenda A. Wilson

**Affiliations:** 1 Department of Microbiology, School of Molecular and Cellular Biology, University of Illinois at Urbana-Champaign, Urbana, Illinois, USA; University of Oklahoma Health Sciences Center, Oklahoma City, Oklahoma, USA

**Keywords:** bacterial toxin, toxin evolution, *Escherichia coli*, enzyme reaction, substrate specificity, protein structure-function, G proteins, mammalian signaling

## Abstract

The cytotoxic necrotizing factor (CNF) family of AB-type bacterial protein toxins catalyze two types of modification on their Rho GTPase substrates: deamidation and transglutamination. It has been established that *E. coli* CNF1 and its close homolog proteins catalyze primarily deamidation and *Bordetella* dermonecrotic toxin (DNT) catalyzes primarily transglutamination. The rapidly expanding microbial genome sequencing data have revealed that there are at least 13 full-length variants of CNF1 homologs. CNFx from *E. coli* strain GN02091 is the most distant from all other members of the CNF family with 50%–55% sequence identity at the protein level and 0.45–0.52 nucleotide substitutions per site at the DNA level. CNFx modifies RhoA, Rac1, and Cdc42, and like CNF1, activates downstream SRE-dependent mitogenic signaling pathways in human HEK293T cells, but at a 1,000-fold higher EC_50_ value. Unlike other previously characterized CNF toxins, CNFx modifies Rho proteins primarily through transglutamination, as evidenced by gel-shift assay and confirmed by MALDI mass spectral analysis, when coexpressed with Rho-protein substrates in *E. coli* BL21 cells or through direct treatment of HEK293T cells. A comparison of CNF1 and CNFx sequences identified two critical active-site residues corresponding to positions 832 and 862 in CNF1. Reciprocal site-specific mutations at these residues in each toxin revealed hierarchical rules that define the preference for deamidase versus a transglutaminase activity in CNFs. An additional unique Cys residue at the C-terminus of CNFx was also discovered to be critical for retarding cargo delivery.

**IMPORTANCE:**

Cytotoxic necrotizing factor (CNF) toxins not only play important virulence roles in pathogenic *E. coli* and other bacterial pathogens, but CNF-like genes have also been found in an expanding number of genomes from clinical isolates. Harnessing the power of evolutionary relationships among the CNF toxins enabled the deciphering of the hierarchical active-site determinants that define whether they modify their Rho GTPase substrates through deamidation or transglutamination. With our finding that a distant CNF variant (CNFx) unlike other known CNFs predominantly transglutaminates its Rho GTPase substrates, the paradigm of “CNFs deamidate and DNTs transglutaminate” could finally be attributed to two critical amino acid residues within the active site other than the previously identified catalytic Cys-His dyad residues. The significance of our approach and research findings is that they can be applied to deciphering enzyme reaction determinants and substrate specificities for other bacterial proteins in the development of precision therapeutic strategies.

## INTRODUCTION

Cytotoxic necrotizing factor 1 (CNF1) is a 110 kDa AB-type protein toxin produced by certain pathogenic strains of *Escherichia coli* that activates members of the Rho family of small GTPases (Rho, Rac, and Cdc42) involved in cytoskeletal and mitogenic signaling (1). CNF1-producing *E. coli* strains are often associated with uropathogenic infections (UPEC strains) (2–4), neonatal meningitis (NTEC strains) (5–9), some diarrheal infections (10–12), and septicemia (13–15). More recently, the presence of the *cnf1* gene has been found in an expanding multidrug-resistant group of extraintestinal pathogenic *E. coli* ST131 (ExPEC) strains, where it confers a competitive colonization advantage in the gut (16). Because CNF1 action results in constitutive activation of Rho GTPases and their downstream signaling pathways, the potential role of CNF1 in cancer progression has been noted (17–22). On the other hand, CNF1 has been explored as a Rho-protein modulator or biologic cargo-delivery system for a number of diverse applications (1, 23), including serving as a vaccine adjuvant (24–26), a bacterial toxin-inspired drug delivery (BTIDD) platform (27–29), a therapeutic tool for pain control (30), and an antineoplastic agent for brain tumors (31–34). CNF1 has also been tested for use in neurodegenerative therapy (35–38) and in learning and memory enhancement (36, 39, 40).

CNF1 activates Rho GTPases by deamidation of the active-site Gln63 in RhoA (41, 42) or Gln61 in Rac1 and Cdc42 (43, 44). Like CNF1, its close homolog CNF2 (85% amino acid sequence identity) activates RhoA and Rac1, and to a lesser extent Cdc42 (45). By contrast, the CNFy homolog (65% shared identity) from *Yersinia pseudotuberculosis* preferentially activates Rho variants (RhoA, RhoB, and RhoC) (46, 47) and only moderately activates Rac1 and Cdc42 (48). CNF3 also acts on all three substrates but prefers RhoA (49). The modular interchangeability of the Gln-deamidase cargo and cargo-delivery domains of the CNF1, CNF2, CNF3, and CNFy variants has been demonstrated and exploited to gain insights regarding the protein determinants that define both the efficacy of cytosolic delivery of cargo and the subsequent intracellular activity (29). In previous studies, comparative analysis of a series of chimeras and selected point mutations between CNF3 and CNFy identified two residues within the putative translocation domain of the delivery vehicles that differentially modulate endosomal escape by the toxins (28).

**Fig 1:**
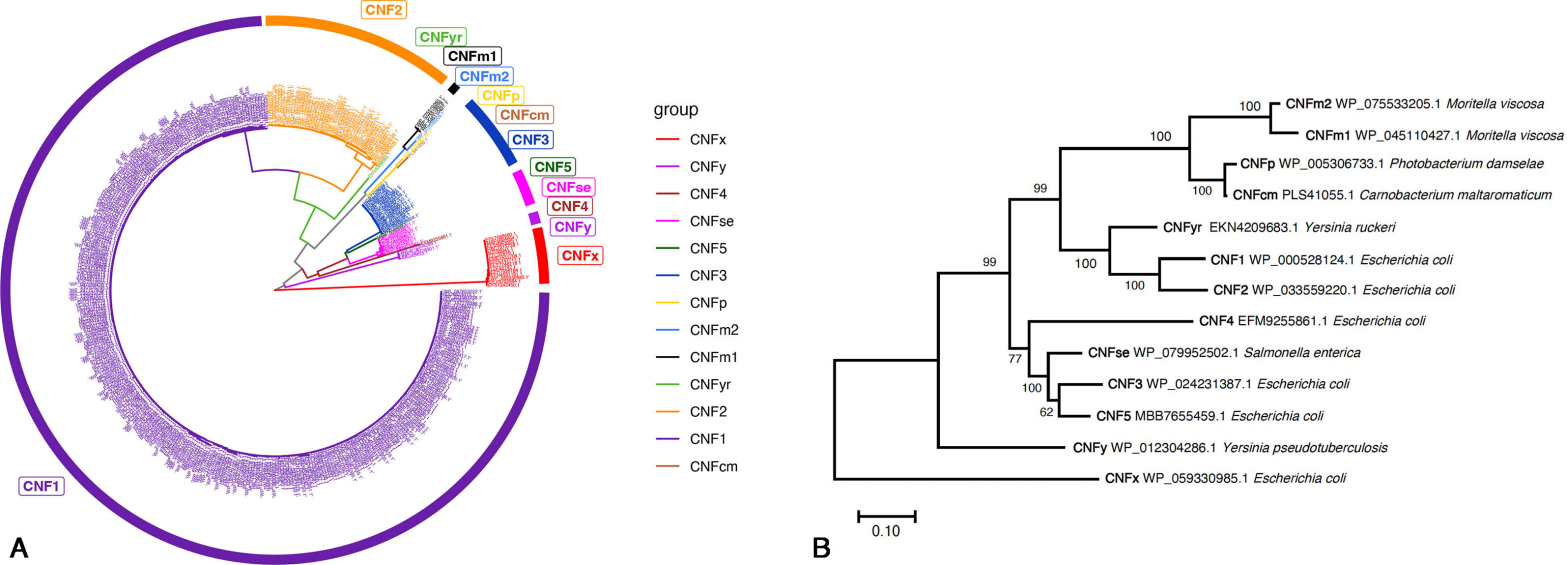
Phylogenetic relationship of full-length CNF toxins. (**A**) Shown is a phylogenetic tree of 435 full-length CNF sequences from the NCBI Protein Database, with accession numbers indicated, generated using raxmlHPC and ggtree in R. (**B**) Shown is a phylogenetic tree of the 13 representative full-length CNF toxins, with accession numbers and source bacteria indicated, generated using MEGA 11 (61).

Over 435 unique full-length CNF toxin sequences clustering into 13 distinct clades can be identified in the NCBI protein database (Fig. 1A). Representatives of each clade, shown in Fig. 1B, have been isolated from a variety of pathogenic bacteria (20), including from *E. coli* (CNF1-CNF5, CNFx), *Salmonella enterica* (CNFse), *Photobacterium damselae* (CNFp), *Moritella viscosa* (CNFm1, CNFm2), *Carnobacterium maltaromaticum* (CNFcm), *Y. pseudotuberculosis* (CNFy), and *Yersinia ruckeri* (CNFyr). The cargo-delivery vehicles of these full-length CNF toxins share homology with the N-terminus of *Pasteurella multocida* toxin (PMT-N) (50, 51), which can deliver not only its cargo but also other types of cargo (27, 52). The CNF1-like catalytic domains have been found in many bacterial genomes associated with a wide range of different cargo-delivery systems (20, 53). However, only a small number of these putative CNF1-like catalytic domain-containing proteins have been studied and confirmed to exhibit deamidase activity. Among these are the T3SS effector VopC from *Vibrio parahaemolyticus* (54), the *Burkholderia pseudomallei* lethal factor 1 BLF1 (55, 56), the T3SS effector protein HopBJ1 from *Pseudomonas syringae* (57), the T6SS effector MIX-CNF from *Vibrio proteolyticus* (58), and the dermonecrotic toxin DNT from *Bordetella* species (51, 59, 60).

DNT, which shares some sequence homology in its C-terminal catalytic domain with CNF1 but none in its N-terminal delivery vehicle, targets the same active-site Gln residues in Rho GTPases as CNF1 (59, 60). Unlike CNF1, which preferentially deamidates its Rho-protein substrates, DNT preferentially transglutaminates them (60, 62, 63). However, in the presence of an overabundance of putrescine, spermidine, spermine, Lys, or other primary amines, CNF1 can also catalyze Rho-protein transglutamination, while in the absence of primary amines, both CNF1 and DNT catalyze deamidation (44, 62–65). In each case, deamidation and transglutamination resulted in constitutive activation of the Rho protein substrates through inhibition of the intrinsic GTPase activity and Rho-GAP-stimulated GTP hydrolysis. The cellular signaling effects of deamidation versus transglutamination were not distinguished in these earlier studies, although another study reported different cellular phenotypes for CNF1 versus DNT action on HEp-2 and Swiss 3T3 cells (66). To date, the general consensus has been that in cells CNF1, and its full-length homologs preferentially deamidate their Rho-protein substrates, while DNT predominantly transglutaminates them, leading in both cases to activation of Rho signaling pathways (67–69). Efforts aimed at identifying the active-site structural features that discriminate between the two reaction mechanisms have been inconclusive based on a direct comparison of CNF1 with DNT (66).

The availability of 13 distinct CNF homologs with high sequence identity, yet differential activity profiles, provides a useful system for investigating determinants that discriminate substrate and reaction mechanism specificities among the CNF variants. We noted that the phylogenetically most distant full-length CNF homolog (CNFx) (Fig. 1), identified in *E. coli* GN02091, shared only 50%–55% amino acid sequence identity with all the other CNFs (54% with CNF1, 55% with CNFy) and a corresponding DNA distance of 0.52 and 0.45 nucleotide substitutions per site. Because the sequence of CNFx was so distant from that of the other CNF toxins, we considered that CNFx might have a different substrate or reaction mechanism preference. We found that CNFx also modifies RhoA in gel-shift assays when co-expressed with RhoA in *E. coli*. Surprisingly, unlike the other well-studied CNFs (CNF1, CNF2, CNF3, and CNFy), CNFx caused a downward gel-shift of the RhoA protein band similar to that reported for DNT-modification of RhoA.

Here, we report a detailed study of CNFx, and through comparison of CNFx with CNF1 using reciprocal mutations, we deciphered two critical active-site residues and a set of hierarchical rules for the CNFs that differentiate between deamidation and transglutamination of their Rho-protein substrates. Following these rules, a single site mutation of DNT converted the DNT transglutaminase into a deamidase. We also identified a critical C-terminal Cys residue that retards cargo delivery and contributes to the higher EC_50_ value of CNFx compared to the other CNFs.

## RESULTS

### Bioinformatic analysis of *cnfx* gene-containing genomes revealed that the *cnfx* gene resides on a putative 153.5 kb conjugative plasmid

The full-length *cnfx* gene coding for Identical Protein Groups (IPG) WP_059330985.1 was identified in 63 *E. coli* genome assemblies of isolates predominantly from cattle or cattle-associated environments, with a few also from human clinical samples. Partial *cnfx* gene fragments were identified in an additional 29 assemblies. Several large *cnfx* gene-containing contigs were found to have identical sequences in the head and tail regions, suggesting that the sequences could be wrapped around to form a circle of about 153.5 kb. An Artemis Synteny plot of four of the largest *cnfx* gene-containing contigs (Fig. S1A) showed high collinearity among each other, albeit with different breaking points, consistent with a circular plasmid structure. Sequence analysis of the *cnfx* gene-containing contigs also revealed the presence of a *repA* gene and conjugal transfer *tra* genes, confirming that the *cnfx* gene was harbored on a putative 153.5 kb conjugative plasmid in *E. coli*. All *cnfx* gene-containing contigs from 92 shotgun genome assemblies aligned to this hypothetical plasmid with >97% coverage and 0.99 identity. A BRIG ring plot of 15 selected *cnfx* gene-containing contig assemblies (Fig. S1B) further illustrates the strong similarity among these putative plasmids. A comprehensive metadata table for *cnfx* gene-containing *E. coli* genomes is included in the supporting material as Table S1. The earliest isolate was recorded in 1980. It is notable that 22 assemblies are of the mlst4382/O61:H16 serotype and 10 are of the mlst4173/O79:H2 serotype, and 52 of the 92 assemblies also harbor Shiga toxin-like (*stx*) genes, with 43 being stx1-S.-sonnei-CB788 (70). Nearly half of the genomes harbored antibiotic resistance genes, and many appeared to be multidrug resistant.

### CNFx exhibits reduced SRE reporter activity compared to CNF1, CNF3, and CNFy

**Fig 2:**
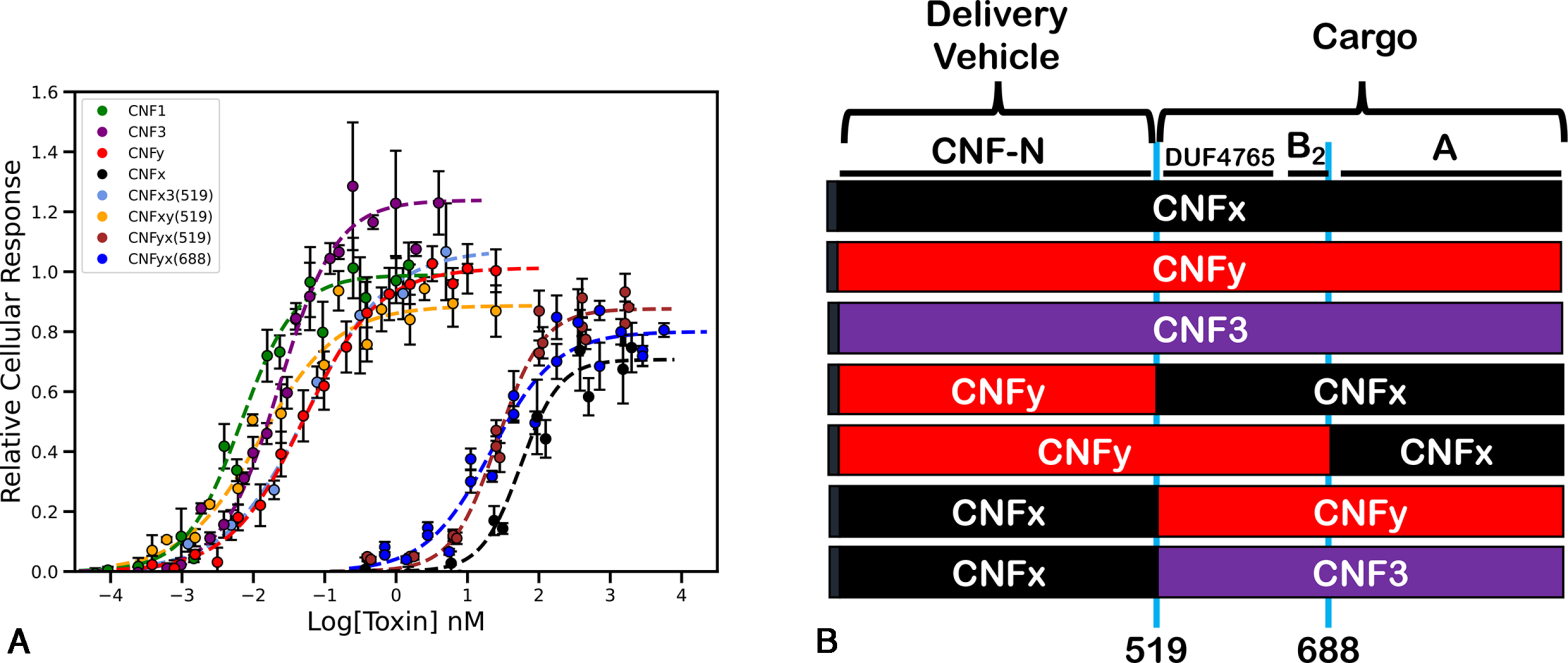
Comparison of cellular activities of wild-type CNF1, CNF3, CNFy, and CNFx and chimeras of CNFx with CNF3 or CNFy. (**A**) Cellular dose-response curves of wild-type CNF1, CNF3, CNFy, and CNFx toxins and chimeric CNFx3(519), CNFxy(519), CNFyx(519), and CNFyx(688) toxins. HEK293T cells transfected with SRE-luciferase gene-reporter plasmids were treated with the indicated toxin at the indicated concentration for 6 h. Cells were then lysed and analyzed for cellular response by dual SRE-luciferase assay, as described in Materials and Methods. The data points shown are the mean values for that specified dose from four independent repeats performed in triplicate. Coomassie-stained SDS-PAGE analysis of purified recombinant proteins of wild-type CNF1, CNF3, CNFy, and CNFx toxins and chimeric toxins CNFx3, CNFxy, and CNFyx using the 519 joining site and CNFyx using the 688 joining site are shown in Fig. S2A. Corresponding scatter plots with all data points used to derive the best-fit lines and mean values are shown in Fig. S2B. (**B**) Schematic diagram depicting the wild type and chimeric toxin constructs generated to test swappability of domains among CNF3, CNFy, and CNFx, where the functional domains (delivery vehicle and cargo) and joining the site (position 519 or 688) of the CNF wild-type and chimeric proteins are indicated. Black = CNFx, red = CNFy, purple = CNF3, A = catalytic domain, B2 = location of the putative secondary binding domain in CNF1, DUF4765 = pfam motif, CNF-N = N-terminus of CNF with homology to PMT-N delivery vehicle.

All previously characterized CNF toxins are known to induce mitogenic signaling as measured in an SRE-luciferase reporter gene assay (29). Like CNF1, CNF3, and CNFy, we found that CNFx also has saturable cellular SRE-response activity in HEK293T cells (Fig. 2A; Fig. S2), but at a much higher EC_50_ value (1,000-fold higher than CNFy) (Table 1), suggesting that CNFx has a much-reduced cargo-delivery efficiency compared to the other CNFs.

**TABLE 1:** Efficiency of cargo delivery by wild-type, chimeric, and site-specific CNF toxins

Toxin	EC_50_ value (nM)	Toxin	EC_50_ value (nM)
CNF1	0.007 ± 0.005	CNF1 (N862E)	0.067 ± 0.009
CNF3	0.023 ± 0.01	CNF1 (R832H)	0.123 ± 0.023
CNFy	0.05 ± 0.03	CNF1 (R832N)	0.026 ± 0.011
CNFx	55.7 ± 2.4	CNF1 (R832N, N862E)	0.065 ± 0.005
		CNF1 (R832H, N862E)	0.046 ± 0.014
CNFx3 (519)	0.058 ± 0.02	CNFx (E857N)	23.3 ± 2.4
CNFxy (519)	0.012 ± 0.011		
CNFyx (519)	26.3 ± 3.6	CNFx (E857N, C1005S)	1.45 ± 0.11
CNFyx (688)	21.4 ± 3.7	CNFx (C1005S)	1.22 ± 0.04

### The N terminus of CNFx can efficiently deliver CNF3 and CNFy cargo

To determine whether the N-terminal delivery vehicle or the cargo of CNFx is responsible for the observed decreased cargo-delivery efficiency, a series of chimeric toxins between CNFx and CNF3 or CNFy was generated using the joining site corresponding to the position at 519 or 688 in CNF1, as depicted in Fig. 2B. These two positions were previously shown to be productive joining sites among the CNF1, CNF2, CNF3, and CNFy proteins (28, 29). As shown in Fig. 2A and summarized in Table 1, using the 519 joining site, we found that CNFx3 (with N-terminal CNFx delivery vehicle plus C-terminal CNF3 cargo) was only threefold less efficient at delivering CNF3 cargo than the wild-type CNF3 protein. Interestingly, CNFxy (with N-terminal CNFx delivery vehicle plus C-terminal CNFy cargo joined at the 519 site) was fourfold more efficient at delivering CNFy cargo than the wild-type CNFy. Attempts to purify the chimeras of CNF3x at either joining site were unsuccessful. Unexpectedly, using the delivery vehicle of CNFy to deliver CNFx cargo (CNFyx) only improved the efficiency of delivery by about twofold, regardless of whether the 519 or 688 site was used to generate the chimeras. This suggests that the CNFx cargo itself might hamper its delivery efficiency.

### CNFx transglutaminates Rho proteins like DNT

**Fig 3:**
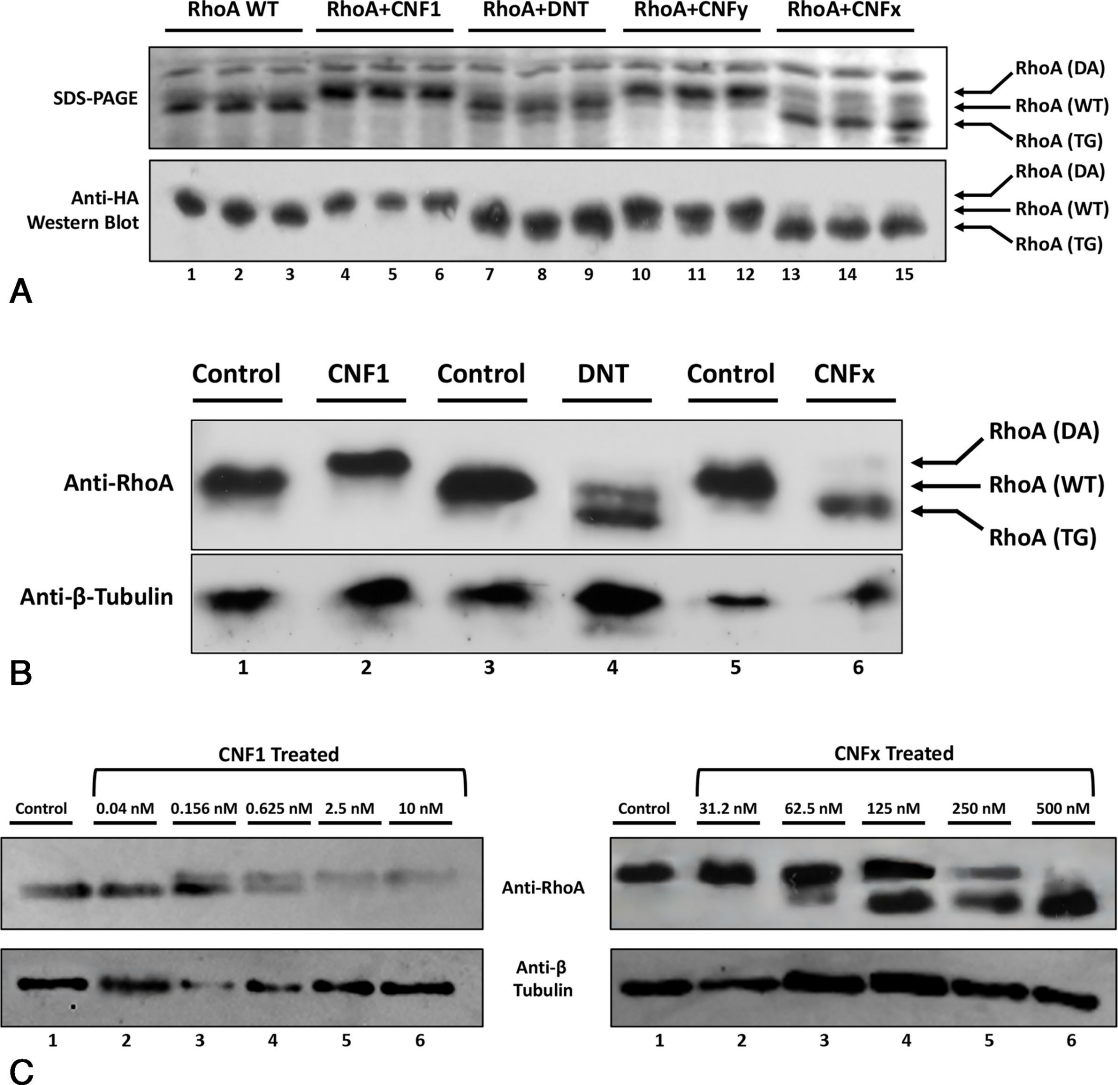
Comparison of RhoA protein modification by CNF1, CNFy, CNFx, and DNT. (**A**) Gel-shift assay of RhoA expressed alone or coexpressed with CNF1, DNT, CNFy, or CNFx toxin in *E. coli* BL21 cells. Bacterial co-expression of recombinant RhoA alone or with the indicated wild-type toxin was performed in triplicate, as described in Materials and Methods. Cell lysates were analyzed by Coomassie-stained SDS-PAGE gel (upper panel) and by western blot using anti-HA antibodies (lower panel). A mobility gel shift of the RhoA band upwards relative to the unmodified RhoA control (WT) indicates deamidation (DA), while a shift downwards indicates transglutamination (TG). (**B**) Gel-shift assay of cell lysates from HEK293T cells treated with 1.5 nM CNF1, 250 nM DNT, or 500 nM CNFx toxin. Transfected HEK293T cells expressing RhoA were treated without (control) or with the indicated toxin in DMEM, as described in Materials and Methods. Cell lysates were separated by SDS-PAGE and analyzed by western blot using first anti-RhoA antibodies (upper panel), then anti-β-tubulin antibodies as loading control (lower panel). (**C**) Representative dose response of RhoA modification by wild-type CNF1 (left panels) and CNFx (right panels) toxins in HEK293T cells using gel-shift assays. Shown are western blots using anti-RhoA antibodies (upper panel) or anti-β-tubulin antibodies (lower panel) of cell lysates from HEK293T cells treated with the indicated toxin concentrations. Additional repeats are shown in Fig. S3A and B, along with the scatter plot used for quantification to determine their respective EC_50_ values for Rho modification (Fig. S3C).

Previous studies have demonstrated that a mobility gel shift of the RhoA protein band upwards relative to the unmodified RhoA control indicates deamidation (41, 42), while a gel shift downwards indicates transglutamination (60, 62, 65). To investigate whether CNFx catalyzes a similar modification of RhoA as the CNFs, HA-tagged RhoA was expressed alone or coexpressed with CNFx, CNF1, CNFy, or DNT in *E. coli* BL21 cells. The resulting reaction profiles were analyzed via SDS-PAGE and western blot using anti-HA antibodies. As summarized in Fig. 3A, RhoA protein coexpressed with CNF1 or CNFy showed an upward gel shift corresponding to deamidation, while RhoA coexpressed with DNT showed a downward shift corresponding to transglutamination. RhoA coexpressed with CNFx showed a downward shift similar to that observed for RhoA coexpressed with DNT.

To confirm that CNFx exhibits the same Rho-modifying activity in mammalian cells, HEK293T cells transiently expressing recombinant RhoA were treated with CNF1, CNFx, or DNT. SDS-PAGE and western blot analyses showed similar mobility shifts for RhoA as those obtained for the bacterial coexpression assays, where CNF1 treatment caused an upward shift of RhoA while CNFx and DNT caused a downward shift (Fig. 3B). These findings confirmed that RhoA is a substrate of CNFx and that CNFx preferentially transglutaminates RhoA in both bacterial and mammalian cells. This further supports that the CNFx-mediated SRE response observed in Fig. 2A involves Rho-protein signaling. Both CNF1 and CNFx displayed saturable, dose-dependent modification of RhoA in HEK293T cells, according to the gel shift assay (Fig. 3C; Fig. S3A and B). However, as expected, the observed EC_50_ values in the gel-shift assay (Fig. S3C), which involved overexpression of RhoA protein, were higher than those observed in the SRE dose-response assay, which relied on endogenous levels of the Rho proteins.

**Fig 4:**
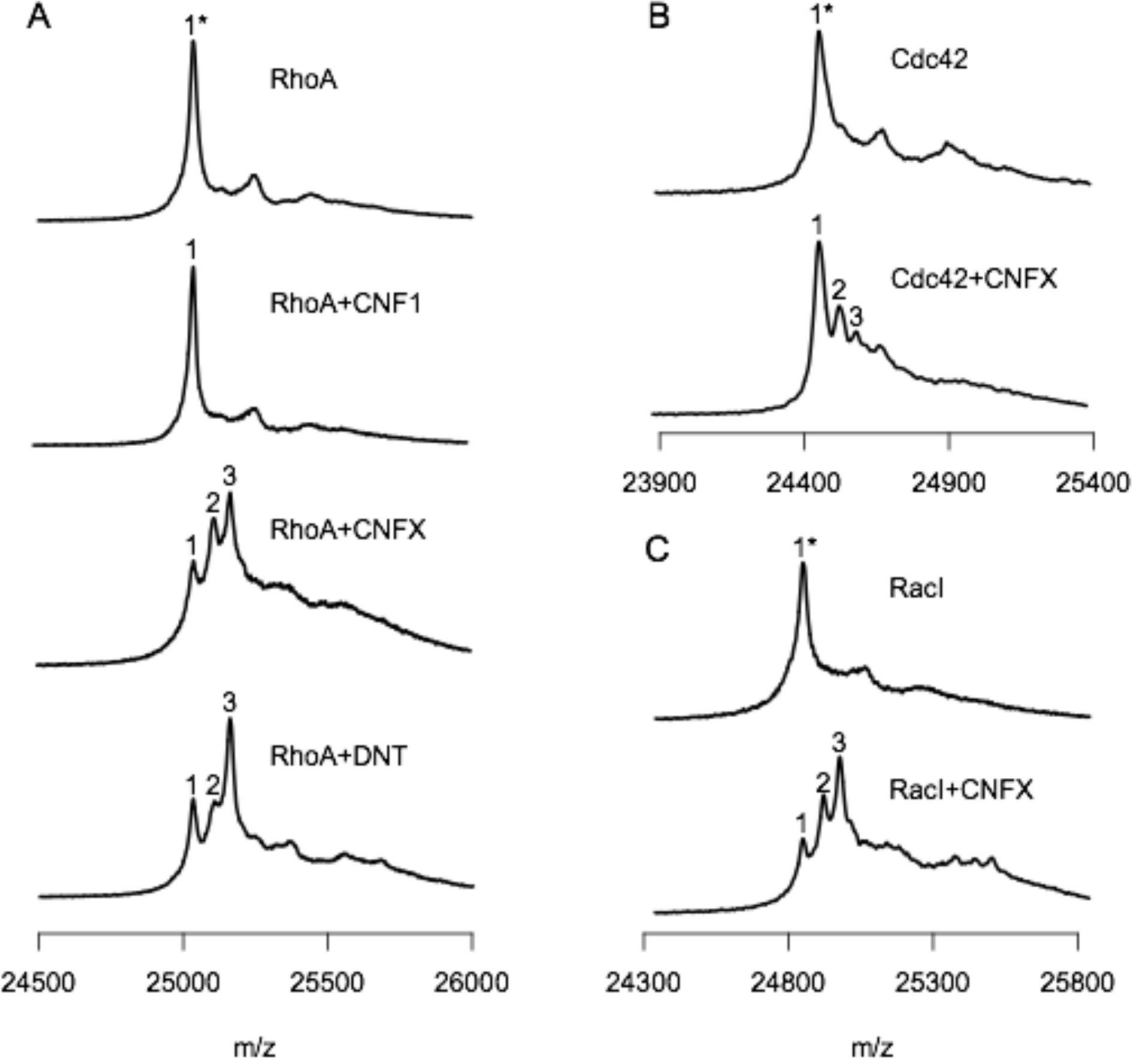
Modification of Rho proteins (RhoA, Cdc42, and Rac1) by CNF1, CNFx, and DNT. MALDI mass spectral analysis of RhoA, Cdc42, and RacI coexpressed in *E. coli* BL21 cells with CNF1, CNFx, or DNT was performed on a Bruker Autoflex Speed LRF MALDI, as described in Materials and Methods. The m/z values shown are uncorrected. (**A**) MALDI mass spectra of RhoA expressed alone (top panel), or coexpressed with CNF1 (second panel), CNFX (third panel), or DNT (bottom panel). (**B**) MALDI mass spectra of Cdc42 expressed alone (upper panel) or coexpressed with CNFx (lower panel). (**C**) MALDI mass spectra Rac1 expressed alone (upper panel) or coexpressed with CNFx (lower panel). The intensities of all spectral plots were normalized to each other based on the highest peak of each plot. 1*, unmodified substrate; 1, deamidated substrate; 2, substrate transglutaminated with putrescine; 3, substrate transglutaminated with spermidine or Lys.

To confirm the transglutaminase activity of CNFx on RhoA and possibly on the other known CNF targets Cdc42 and Rac1, the recombinant His_6_-tagged G-proteins were expressed alone or coexpressed with CNF1, CNFx, or DNT in *E. coli* BL21 cells, and then purified by nickel-affinity and anion-exchange chromatography. The resulting G-proteins were then subjected to analysis by MALDI mass spectrometry (Fig. 4). The expected mass size for unmodified His_6_-tagged RhoA is 25,034 Da. CNF1 deamidation of RhoA yields a single Dalton increase in the molecular size of the RhoA protein (25,035 Da). However, the MALDI mass spectral peak for the Q63E change was indistinguishable from the unmodified RhoA peak (Fig. 4A). While DNT also deamidates RhoA to yield Q63E (60), it preferentially transglutaminates RhoA with Lys or other primary amines such as putrescine and spermidine (64). These modifications of RhoA resulted in three observable peaks detected in the MALDI mass spectra of RhoA coexpressed with DNT. Based on the observed *m*/*z* of the peaks, the first peak (labeled as 1* or 1) represents the unmodified or deamidated RhoA, respectively. The second peak (labeled as 2) represents a transglutamination of RhoA with putrescine (∆71 *m*/*z*). The third and most prominent peak (labeled as 3) is most likely transglutamination of RhoA with Lys (∆129 *m*/*z*) or spermidine (∆128 *m*/*z*)—the resolution of the spectra was insufficient to discriminate between the two modifications. The MALDI mass spectra of RhoA coexpressed with CNFx displayed similar *m*/*z* peaks as those for RhoA coexpressed with DNT but with different ratios of peak intensities, supporting that CNFx modification of RhoA involves transglutamination, with a substrate preference for putrescine over Lys or spermidine. The CNFx-modified Cdc42 (Fig. 4B) and Rac1 (Fig. 4C) also displayed three observable peaks representing unmodified or deamidated protein (peak labeled as 1* or 1) and transglutaminated protein (peaks labeled 2 and 3) with similar observed ∆*m*/*z* values as observed for DNT- or CNFx-modified RhoA. The most prominent peak of the CNFx-modified Cdc42 corresponded to the size of unmodified or deamidated Cdc42, indicating that perhaps CNFx has either a lower affinity for Cdc42 or it preferentially deamidates Cdc42. By contrast, CNFx-modified Rac1 had two prominent peaks (labeled 2 and 3) larger than the unmodified or deamidated Rac1 peak (labeled 1* or 1), showing a preference for transglutamination of Rac1.

### Identification of two residues near the catalytic active site that define deamidation versus transglutamination in CNF variants

**Fig 5:**
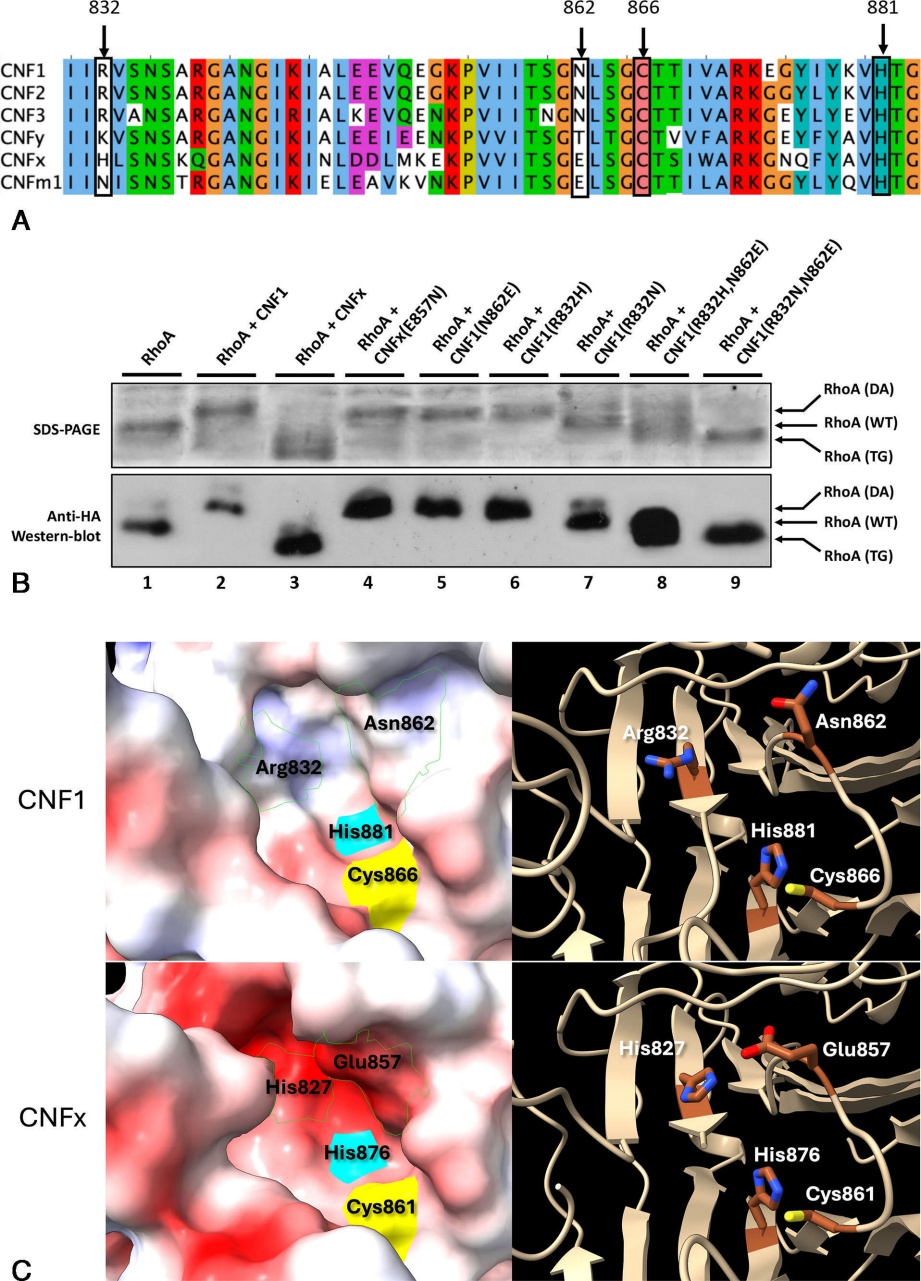
Comparison of the active site sequences, structures, and activities of the C-terminal catalytic domains of the CNF toxins. (**A**) The amino acid sequences around the catalytic pocket of CNF1, CNF2, CNF3, CNFy, CNFx, and CNFm1 were aligned using Muscle and visualized using Jalview to color the amino acid residue sequences in Clustal format. The conserved catalytic residues C866 and H881 of the CNF toxins are indicated. Other active-site residues corresponding to the number in CNF1 at positions 832 and 862 are also indicated. (**B**) Gel-shift assay of RhoA expressed alone or coexpressed with wild-type CNF1 or CNFx or with point mutants CNF1 (R832H), CNF1 (R832N), CNF1 (R832H, N862E), CNF1 (R832N, N862E), and CNFx (E857N) in *E. coli* BL21 cells, performed and analyzed as described in Materials and Methods. Cell lysates were analyzed by Coomassie-stained SDS-PAGE gel (upper panel) and by western blot using anti-HA antibodies (lower panel). SDS-PAGE analysis of the recombinant wild-type and point mutants of CNF1 and CNFx expressed and purified from *E. coli* BL21 are shown in Fig. S4A. (**C**) Closeup view of structural models of the active sites of CNF1 and CNFx. Models were generated in Modeler using the known crystal structures containing the CNFy and CNF1 C-terminal domain (6YHK, 6YHM, 6YHN, and 1HQ0 pdb), as described in the Materials and Methods. Visualizations were performed in ChimeraX. The catalytic Cys-His dyad and the mutated residues are indicated. In the electrostatic surface models, the catalytic Cys is yellow, and the catalytic His is cyan. Structural models of the complete C-terminal sequences of the CNF proteins (CNF1, CNF2, CNF3, CNFy, CNFx, and CNFm1) from the start of the catalytic domains (corresponding to position 735 in CNF1) generated using Modeller or SWISS-Modeller are shown in Fig. S5.

Since CNFx is the first full-length CNF toxin that preferentially transglutaminates its small G-protein targets rather than deamidates them, we considered whether comparison of the active site of CNFx with other full-length CNF homologs might provide insights regarding the determinants driving deamidation versus transglutamination. In line with this, we compared the C-terminal catalytic sequences of CNFx with the corresponding sequences of previously characterized CNF1, CNF2, CNF3, and CNFy (Fig. 5A), which are known to preferentially deamidate RhoA. We thereby identified a residue corresponding to position 862 in CNF1 that is near the catalytic Cys (C866 in CNF1) and His (H881 in CNF1) and that is different in CNFx from the others. CNFx contains a Glu (E857) at the equivalent site, while CNF1/2/3 have an Asn (N862 in CNF1 and CNF2, N861 in CNF3) and CNFy has a Thr (T862). Mutation of this residue in CNFx to Asn (E857N) resulted in deamidation of RhoA when coexpressed in *E. coli* BL21 (Fig. 5B, lane 4). However, the reciprocal change in CNF1 (N862E) had no effect on CNF1-catalyzed deamidation of RhoA (Fig. 5B, lane 5). These findings suggest that there are additional residues defining the two possible enzymatic outcomes: deamidation versus transglutamination.

An additional active-site residue at position 832 in CNF1 was identified through comparison of homology models of the CNF C-terminal catalytic domains generated using the crystal structures of CNF1 and CNFy (Fig. 5C; Fig. S5). The residue at position 832 in CNF1, which is a His in CNFx, but an Arg in CNF1, CNF2, and CNF3, and a Lys in CNFy, was near the residue at position 862 in the active site of these models, indicating that it might affect the reaction outcome of the toxins. Mutation of this residue to His in CNF1 (R832H) resulted in no apparent loss of deamidation activity (Fig. 5B, lane 6). By contrast, mutation at both sites in CNF1 (R832H, N862E) resulted in not only loss of deamidation activity but instead gain of transglutamination activity (Fig. 5B, lane 8).

**Fig 6:**
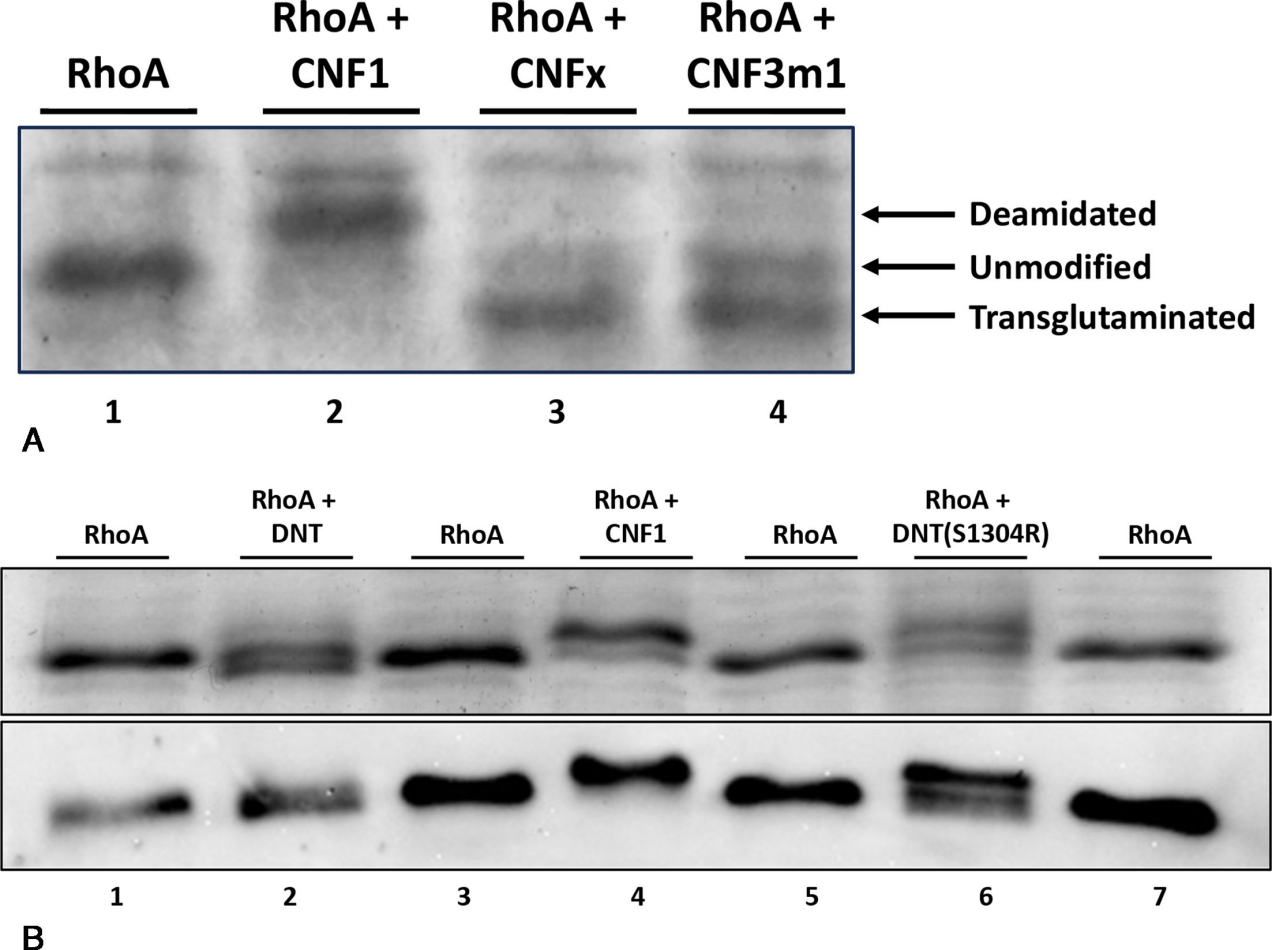
RhoA modification activities of CNF1, CNFx, and other CNF-like toxins CNF3m1, and wild-type and mutant DNT. (**A**) Gel-shift assay of RhoA expressed alone or coexpressed with wild-type CNF1, CNFx, or CNF3m1 in *E. coli* BL21 cells, performed and analyzed as described in Materials and Methods. Cell lysates were analyzed by Coomassie-stained SDS-PAGE gel. (**B**) Gel-shift assay of RhoA expressed alone or coexpressed with wild-type CNF1, wild-type DNT, or point mutant DNT (S1304R) in *E. coli* BL21 cells, performed and analyzed as described in Materials and Methods. Cell lysates were analyzed by Coomassie-stained SDS-PAGE gel (upper panel) and by western blot using anti-HA antibodies (lower panel). Additional repeats are shown in Fig. S6B.

Among the CNF variants, CNFm1 has an Asn at position 832 (Fig. 5A). Again, even though a single mutation at this site in CNF1 (R832N) retained partial deamidase activity (Fig. 5B, lane 7), mutation at both sites in CNF1 (R832N, N862E) completely converted the double mutant into a transglutaminase (Fig. 5B, lane 9). Since these two positions in the CNF1 (R832N, N862E) mutant are the same as those found in both CNFm1 and CNFm2 (Fig. S6A), it is reasonable to anticipate that CNFm1 and CNFm2 variants would also possess transglutaminase activity. This was confirmed by coexpression of chimeric CNF3m1 (comprised of the CNF3 delivery vehicle plus the CNFm1 cargo) with RhoA in *E. coli* BL21 cells (Fig. 6A), which as expected displayed exclusive transglutaminase activity. This finding indicates that both residues at positions 832 and 862 within the catalytic pocket influence the enzymatic activity. Interconverting the residues at these positions can change the preference toward deamidation as in CNF1 (and CNF2, CNF3, and CNFy) or toward transglutamination as in CNFx and CNFm1.

### Mutation of a residue in DNT at position analogous to R832 in CNF1 converts DNT into a deamidase

To determine whether the residues at positions corresponding to 832 and 862 in CNF1 play a role in determining the reaction outcome in the broader CNF family, we considered whether DNT, which is preferentially a transglutaminase, could be converted into a deamidase. Point mutation of the Ser residue at position 1304 (S1304) in DNT that corresponds to R832 in CNF1 (Fig. S6A) to Arg (DNT [S1304R]) switched the activity of DNT from transglutaminase to deamidase when coexpressed with RhoA in *E. coli* (Fig. 6B; Fig. S6B).

### Deamidation versus transglutamination does not account for the large difference in EC_50_ values between CNF1 and CNFx in the SRE cellular response assay

**Fig 7:**
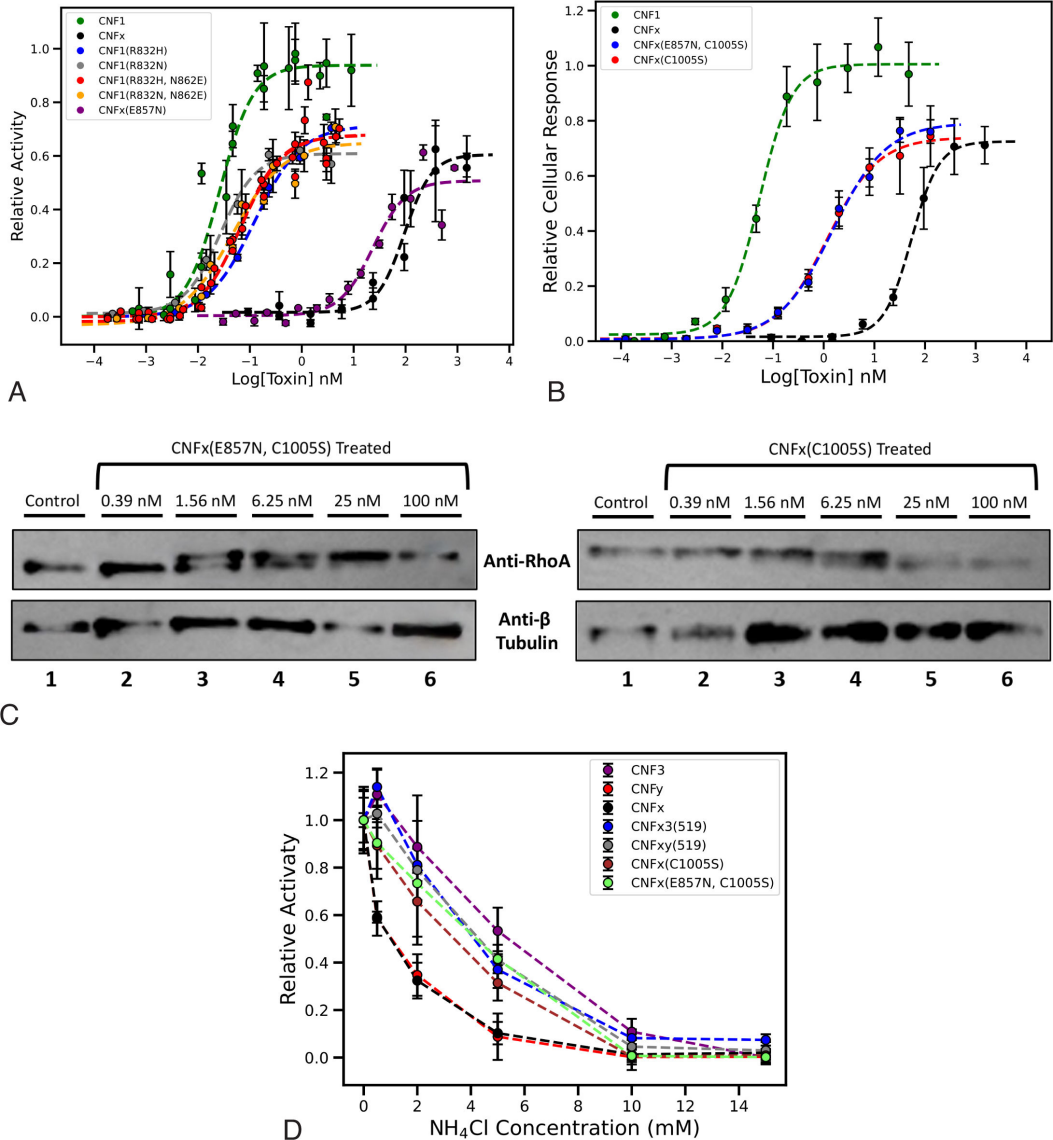
Impact of CNFx Cys mutation (C1005S) on cellular activity, RhoA modification, and sensitivity to endosomal acidification. (**A**) Cellular dose-response curves for wild-type CNF1 and CNFx and the point mutants CNF1 (R832H), CNF1 (R832N), CNF1 (R832H, N862E), CNF1 (R832N, N862E), and CNFx (E857N) in HEK293T cells. Cells were treated with the indicated toxin concentration for 6 h, then lysed and analyzed by SRE-luciferase assay, as described in Materials and Methods. The data points shown are the mean values for that specified dose from three independent repeats performed in triplicate. Corresponding scatter plots with all data points used to derive the best-fit lines and mean values are shown in Fig. S4B. (**B**) Cellular dose-response curves for wild-type CNF1 and CNFx and the point mutants CNFx (C1005S) and CNFx (E857N, C1005S) in HEK293T cells. Cells were treated with the indicated toxin concentration for 6 h, then lysed and analyzed by SRE-luciferase assay, as described in Materials and Methods. Data points shown are the mean values for that specified dose from three independent repeats performed in triplicate. SDS-PAGE analysis of the recombinant wild type and point mutants purified from *E. coli* BL21 are shown in Fig. S8A. Corresponding scatter plots with all data points used to derive the best-fit lines and mean values are shown in Fig. S8B. (**C**) Representative dose response blots of RhoA modification by CNFx (C1005S) (right panels) and CNFx (E857N, C1005S) (left panels) toxins in HEK293T cells using RhoA gel-shift assays, performed as described in Materials and Methods. Shown are western blots using anti-RhoA antibodies (upper panel) or anti-β-tubulin antibodies (lower panel) of cell lysates from HEK293T cells treated with the indicated toxin concentrations. Additional repeats are shown in Fig. S8C and D, along with the scatter plot used for quantification to determine their respective EC_50_ values for Rho modification (Fig. S8E). (**D**) Sensitivity of wild-type and mutant CNF toxins to endosomal acidification. Shown are dose–response curves to NH_4_Cl treatment of the wild-type CNF toxins (CNF3, CNFy, CNFx), chimeric toxins (CNFx3, CNFxy), and point mutants [CNFx (C1005S), CNFx (E857N, C1005S)] in the SRE-luciferase assay, as described in Materials and Methods. HEK293T cells were treated with NH_4_Cl for 30 min prior to treatment with the indicated toxins and assayed for SRE cellular response activity after 6 h. Relative activity indicates the fold activation compared with no-inhibitor treatment. Data points shown are the mean values for that specified dose from three independent repeats performed in triplicate. Corresponding scatter plots with all data points used to derive the best-fit lines and mean values are shown in Fig. S8F.

To determine whether transglutaminase versus deamidase activities was responsible for the observed reduced efficiency of cargo delivery for CNFx, we compared the wild-type and point mutants of CNF1 and CNFx in the cellular SRE-reporter dose-response assay. Interestingly, conversion of CNF1 into a transglutaminase through double mutation at the two pivotal positions, CNF1 (R832H, N862E) or CNF1 (R832N, N862E), only increased the EC_50_ value in the SRE cellular response assay by less than 10-fold, while conversion of CNFx into a deamidase, CNFx (E857N), only reduced the EC_50_ value by less than 10-fold (Fig. 7A; Table 1). There was likewise no significant difference in the time course of the SRE cellular response between the wild-type and point mutants of the corresponding proteins (Fig. S7). Thus, although the transglutaminase version of the CNF protein has a higher EC_50_ value than the deamidation version of the same protein, there is still a notable 500-fold difference in the EC_50_ values between the CNF1 transglutaminase mutant and the CNFx deamidase mutant (Table 1).

### Removal of a unique C-terminal Cys in CNFx enhances the cargo-delivery efficiency of CNFx and alters CNFx sensitivity to endosomal acidification

In considering possible explanations for the observed differences in EC_50_ values between CNFx and the other CNF proteins, we noted that the delivery vehicle for CNFx is as good as that of CNFy, as observed for the CNFxy and CNFx3 chimeras, and concluded that the CNFx cargo most likely contributed to retardation of cargo delivery, as observed for CNFyx (Fig. 2A). We noticed a unique Cys residue in CNFx at position 1005 that is not present in any of the other CNF toxins. To evaluate the impact of this Cys on the efficiency of cargo delivery by CNFx, this residue was replaced with a Ser residue in both wild-type CNFx (C1005S) and the deamidase version of CNFx (E862N, C1005S). The potency of the two Cys mutants, CNFx (C1005S) and CNFx (E857N, C1005S), are nearly the same, in terms of cargo delivery efficiency in the SRE cellular response assay (Fig. 7B) and for RhoA modification in the gel-shift assay (Fig. 7C; Fig. S8). Removal of this Cys residue enhanced the cargo-delivery efficiency of both mutants over wild-type CNFx by more than 50-fold (Table 1).

For AB toxins like the CNFs, the intoxication process involves receptor-mediated endocytosis, followed by a pH-dependent translocation of the cargo across the endosomal membrane and into the cytosol (68). Among the previously studied CNF toxins, CNF3 and CNFy exhibited the greatest difference in sensitivity to endosomal pH during cargo delivery (28), with CNF3 being the most resistant to acidification inhibitors and escaping the endosome earlier at a higher pH than the more sensitive CNFy. To explore whether sensitivity to endosomal pH might play a role in the lower cargo-delivery efficiency of CNFx, we tested the impact of the endosomal acidification inhibitor NH_4_Cl on the SRE-reporter activity of wild-type CNFx, CNF3, and CNFy, the chimeras CNFx3 (519) and CNFy (519), and the Cys mutants CNFx (C1005S) and CNFx (E857N, C1005S). As shown in Fig. 7D, wild-type CNFx was as sensitive to NH_4_Cl treatment as CNFy; however, CNFx3, CNFxy, CNFx (C1005S), and CNFx (E857N, C1005S) all showed enhanced resistance to NH_4_Cl like CNF3, consistent with improvement of cargo-delivery efficiency.

## DISCUSSION

The widespread prevalence of CNF1-like effector domains associated with multiple forms of cargo-delivery vehicles and the importance of their Rho GTPase targets in multiple cellular signaling pathways make them effective microbial tools for manipulating host cells during infection, but also as cell biology tools for deciphering signaling pathways. The knowledge accumulated so far is an incomplete picture of what toxin protein determinants define the Rho GTPase deamidase versus transglutaminase activity of the CNF proteins and the subsequent impact of that activity on cellular signaling pathways. For these toxins to be applied most effectively as biological tools and therapeutics, it is critical that their biological activities are thoroughly defined and understood. Toward this end, coexpresssion of Rho proteins with CNF1, CNFx, and DNT in *E. coli*, followed by gel-shift assay and mass spectral analysis enabled the discernment of reaction preferences among these toxins. While CNF1 displayed a clear preference for RhoA deamidation, CNFx like DNT showed more transglutamination. With our finding that CNFx acts as a transglutaminase, the paradigm of “CNFs deamidate and DNT transglutaminates” can finally be attributed to clearly defined structural determinants in the toxin active site. Using a series of site-directed mutants of CNF1 and CNFx, we found two critical amino acid residues, corresponding to positions 832 and 862 in CNF1, that dictate the enzymatic activity of deamidase versus transglutaminase, adhering to hierarchical rules exemplified by three scenarios, as summarized in Table 2.

**TABLE 2:** Hierarchical rules defining deamidation versus transglutamination

Scenario	Parent sequence	CNF1 positions 832/862	Enzyme reaction	Reference
Arg/Lys at 832	CNF1	Arg/Asn	Deamidation	This study (41, 42)
	CNF2	Arg/Asn		(45)
	CNF3	Arg/Asn		(49)
	CNFy	Lys/Thr		This study (47)
	CNF1	Arg/Glu		This study
Non-Arg/Lys at 832 + Glu at 862	CNFx	His/Glu	Transglutamination	This study
	CNFm1	Asn/Glu		
	CNF1	His/Glu		
	CNF1	Asn/Glu		
Non-Arg/Lys at 832 + non-Glu at 862	CNFx	His/Asn	Deamidation	This study
	CNF1	Asn/Asn		
	CNF1	His/Asn		

For scenario 1, whenever the active-site residue at position 832 is an Arg or Lys, the enzyme performs predominantly as a deamidase. In the sequences of CNF1, CNF2, and CNF3, the residue at 832 is an Arg and in CNFy it is a Lys (Fig. 5A). The single mutant CNF1 (N862E), which adheres to this rule with an Arg at position 832, performed as a deamidase. For scenario 2, in the absence of this Arg/Lys at position 832, if the residue at position 862 is a Glu, then the enzyme becomes a transglutaminase, as in CNFx which has His at position 832 and Glu at position 862, and in CNFm1 which has Asn and Glu at these two positions, respectively. Double mutants of CNF1, CNF1 (R832H, N862E) and CNF1 (R832N, N862E), likewise demonstrated transglutaminase activity. For scenario 3, in the absence of Arg/Lys at position 832, and without Glu at position 862, as in the case of the single point mutants CNF1 (R832H), CNF1 (R832N), and CNFx (E857N), the enzyme performed as a deamidase. Thus, it can be rationalized that the presence of an Arg or Lys at the active site is likely to prevent the approach of a positively charged polyamine to the active site as a substrate, and so preference will be for hydrolysis (deamidation) rather than transglutamination. When this positively charged barrier is removed, that is, no Arg or Lys at position 832, then the presence of an additional negatively charged Glu facilitates the approach of a positively charged polyamine to the active site and transglutamination can ensue. The absence of the positively charged barrier without the additional negative charge at position 862 is insufficient to catalyze transglutamination in CNF1 homologs, as was observed for the case of the three mutants tested, CNF1 (R832H), CNF1 (R832N), and CNFx (E857N). Consistent with these observations, structural modeling of the CNFs revealed differences in the electronegative charges within the active-site pocket of deamidase-preferring CNFs (CNF1, CNF2, CNF3, and CNFy) versus transglutaminase-preferring CNFx and CNFm1 (Fig. 5C; Fig. S5).

Examination of the 435 full-length CNF toxin sequences (Fig. 1A) revealed that 411 of the 435 sequences have an Arg at position 832 and an Asn at 862, including all the sequences in the CNF1, CNF2, CNF3, CNF4, CNFse, CNFyr, and CNFp clades. All the CNFy sequences have Lys at position 832 and Thr at position 862. Since all these clades have Arg or Lys at position 832, and therefore fall under the dominance of scenario 1 (rule 1), we anticipate that they will have deamidase activity like CNF1, CNF2, CNF3, and CNFy. Even though CNF5 has an Asp at 862, this is likely overridden by the dominant Arg at position 832, and we anticipate it will likewise be a deamidase. On the other hand, CNFx has His and Glu at the respective positions and preferentially carries out transglutamination, and so, it would not fall under the dominance of Arg/Lys for rule 1, but would adhere to scenario 2 (rule 2) with a negative charge at position 862. Since CNFm1 and CNFm2 have Asn and Glu at these respective positions, they would also be expected to be transglutaminases. Consistent with this, we found that CNFm1, like CNFx, also preferentially transglutaminates RhoA (Fig. 6A).

Outside of the full-length CNF family, the C-terminal catalytic domain is observed in a variety of other bacterial proteins (Fig. S9). The activities of most of these sequences have not been studied; the only ones with known activity cluster to the separate CNF, VopC, and DNT clades. Among the CNFs specifically, CNFx and CNFm1, which have preference for transglutaminase activity, cluster separately from each other in both the full-length and C-terminal domain-only trees (Fig. 1). There is also no apparent evolutionary grouping of the transglutaminase versus deamidase activities in the broader C-terminal CNF homolog tree that we can discern from known reported activities or from prediction based on the rules we have posited in this paper.

VopC from *Vibrio parahaemolyticus* is known to carry out deamidation in *E. coli* BL21 cells when coexpressed with Rac1 (54). The VopC used in this study has His and Ala residues at the respective positions corresponding to 832 and 862 in CNF1. This follows the rules for these positions we have laid out here, where in the absence of Arg at 832 and the absence of Glu at 862 (scenario 3/rule 3), the preferred activity is deamidation.

DNT, another CNF catalytic domain-containing homolog outside the full-length CNF clade, is known to carry out primarily transglutamination (60, 62). At the analogous position to 832 in CNF1, there is a Ser (not Arg or Lys) in DNT. This does not contradict the hierarchical rule 1 that requires the presence of Arg or Lys at this position to be a deamidase. Indeed, when this residue (S1304) is changed to Arg (S1304R), DNT becomes a deamidase. However, unlike CNFx or CNFm1, DNT has a Ser and not a negatively charged Glu at the analogous 862 position. We speculate that there might be additional negatively charged residue(s) in DNT that supplement the function of Glu at 862, necessitating a modified scenario 2 for DNT, where other negative charges need to be present nearby to facilitate transglutamination. Consistent with this assessment, the alignment of DNT with CNF homologs (Fig. S6A) shows significant variation in the active-site region. Over half of the residues within the 824–883 active-site region of DNT are different from those found in the CNFs, including several Glu residues that could serve the same function as E862 in the CNFx and CNFm1 transglutaminases and the CNF1 transglutaminase mutants.

As we have shown here, the overall efficiency of toxin delivery by the full-length wild-type toxin variants does not necessarily equate to the same efficiency observed of the individual functional modules comprising the toxin. The full-length CNFx is not an efficient assembly of cargo and delivery vehicle modules like the other CNFs are. However, the N-terminal CNFx delivery vehicle is more efficient than the N-terminus of CNFy at delivering CNFy cargo (fourfold CNFxy versus CNFy), but still not as good as CNF3 at delivering CNF3 cargo (threefold CNF3 versus CNFx3) (Table 1). These findings emphasize the importance of domain compatibility between cargo and delivery vehicle modules among these toxins, as previously observed (29).

We also show the feasibility of modulating the efficiency of cargo delivery through mutation of the cargo. Unlike the other CNFs, CNFx contains a unique Cys residue near the C-terminal end (residues LLCRELL) of its cargo module. Mutation of this Cys residue at position 1005 enhanced the cargo delivery efficiency by 50-fold, regardless of whether the cargo behaved as a deamidase (CNFx [E857N, C1005S]) or a transglutaminase (CNFx [C1005S]). This Cys-containing LLCRELL motif is a potential modulator for therapeutic applications of biological toxins.

Our results demonstrate the power of harnessing the evolutionary relationships by comparing many homologous naturally occurring toxin sequences to tease out overlapping biological functions. Even though CNFx is a less efficient toxin compared to the other CNFs, the discovery of its transglutaminase activity enabled us to connect and discriminate the deamidase and transglutaminase activities of CNF1 and DNT, respectively. In addition to what we have delineated here regarding the chemical reaction specificity of the CNF homologs, a similar approach could be applied toward defining the structural determinants for Rho-protein substrate specificity of CNF1 homologs. This refinement can lead to the development of CNF1 homologs as highly specific Rho GTPase modulators for manipulating biological signaling pathways in precision therapeutic strategies. Similar approaches for comparing other modular bacterial toxins could lead to the realization of additional bacterial toxin-inspired drug delivery (BTIDD) tools (28, 29).

## MATERIALS AND METHODS

### Bioinformatic analysis of CNFx-containing genomes

Genome assemblies containing sequences coding for IPG WP_059330985.1 or partial fragments (>600 aa), 63 and 29 counts respectively, were collected using ncbi-datasets-cli v15.31.2 for bioinformatic analysis. The header region of genomic.gbff files were extracted and filtered using a shell script to generate a metadata file for the *E. coli* isolates (Table S1). Conda packages mlst v2.23.0, serotypefinder v2.0.1, amrfinderplus v3.10.18, and virulencefinder v2.04 were used to identify mlst sequence type, O- and H-antigens, antimicrobial resistance genes, and virulence genes, respectively. Artemis comparison tool (ACT) v18.1.0 and BLAST Ring Image Generator (BRIG) tool v0.95 were used for sequence comparison.

Full-length CNF sequences were identified using the NCBI BLAST tool, where only complete sequences with amino acid lengths of 1009 to 1035 residues were kept (i.e., truncated sequences were removed) and aligned with each other using Muscle5 (71). The full-length sequences were used to generate a phylogenetic tree calculated using the Jones-Taylor-Thorton (JTT) model in RAxML v8 (72) and visualized in R with ggtree v3.10 (73). MEGA 11 (61) was used to calculate and generate a maximum-likelihood phylogenetic tree of the 13 representative full-length CNF toxin sequences by the JJT model with complete deletion of positions with missing residues, 500 bootstrap replications, and the nearest-neighbor-interchange (NNI) ML heuristic method.

For the CNF catalytic domain homology tree, NCBI BLAST was used to identify homologs to the CNF1 C-terminus. Identified sequences were clustered using CD-HIT v4.8.1-2019-0228 with a 95% identity threshold. Representative sequences from each cluster were aligned using NCBI COBALT (74), where sequences without alignment to the CNF1 C-terminus were removed. The COBALT alignment was used for another round of CD-HIT clustering, and 163 representative sequences from each cluster were aligned with COBALT and trimmed to the homologous region of the CNF1 C-terminal sequence. These sequences were then used to generate a phylogenetic tree with RAxML and visualized with R, as described above.

### Construction of genes encoding wild-type and mutant CNF proteins

Recombinant CNF proteins were constructed as previously described (28, 29). The gene encoding full-length CNFx was constructed from three synthetic gBlock gene fragments (Integrated DNA Technologies, Inc.) with specific restriction sites on each end for joining the fragments and cloning into the SuperG vector, a plasmid constructed in our laboratory for high-level His_6_-tagged protein expression in *E. coli*, as described previously (28, 29). The gene encoding the C-terminal cargo of CNFm1 was constructed from a synthetic gBlock gene fragment (Integrated DNA Technologies, Inc.) and then exchanged with the cargo of CNF3 in the full-length CNF3 construct at the 688 joining site to yield the CNF3m1 chimera. For the construction of other CNF chimeras, PCR amplification with overlapping primers was used to generate the joining sites at positions 519 and 688 for swapping functional domains of CNFx with CNF1, CNF3, or CNFy, as well as to generate the single and double amino acid point mutants.

### Purification of CNF proteins

All recombinant CNF proteins were expressed in *E. coli* BL21, and then purified and quantified, as previously described (28, 29). SDS-PAGE analyses of the resulting purified proteins are shown in Fig. S2A, S4A, and S8A.

### Toxin coexpression with Rho proteins in *E. coli* BL21 cells

Plasmids encoding the His_6_-tagged G-protein (RhoA, Rac1, or Cdc42) were transformed alone or co-transformed with a plasmid containing the indicated toxin at a 1:1 ratio into competent *E. coli* BL21 cells. The recombinant His_6_-tagged G-proteins were expressed from pET33 vector plasmids under IPTG-inducible *lac* operon control, while toxins were expressed constitutively from the SuperG plasmids described above. Dual-plasmid-containing *E. coli* BL21 cells were incubated overnight (16–18 h) at 37˚C, then diluted 10-fold into new culture tubes and induced with 100 µg/mL IPTG for 5 h. Cells (1 mL) were harvested via centrifugation and then lysed with 250 µL 5× SDS sample buffer at 95°C for 5 min. Gel-shift assays were performed by subjecting the cell lysates to SDS-PAGE analysis using gels with a tiered lower stack of 12.5% (top) and 15% (bottom) acrylamide. Gels were visualized by Coomassie stain, followed by western blot analysis using mouse anti-RhoA (Santa Cruz Biotechnology, sc-418, lot: B0521) or anti-HA (Invitrogen, ref: 26183, lot: TJ272546) as primary antibodies, goat anti-mouse IgG (Santa Cruz Biotechnology, sc-516102, lot: G0621) as secondary antibodies, and the ECL plus system (GE Life Sciences) for visualization, as previously described (52).

### MALDI mass spectral analysis

Recombinant His_6_-tagged Rho proteins, RhoA, Cdc42, and Rac1, were expressed alone or coexpressed with the indicated toxin in *E. coli* BL21 cells, as described above. The cells were harvested by centrifugation at 4,300 *g*. The cell pellets were resuspended in lysis buffer [phosphate-buffered saline (PBS), pH 7.4, containing 0.5% Triton X-100, 0.3 mg/mL lysozyme (VWR Life Science AMRESCO), 2 mg/mL benzamidine (Thermo Scientific), 0.3 mg/mL phenylmethylsulfonyl fluoride (DOT Scientific), 5 Kunitz units/mL DNase (Thermo Scientific), 10 µg/mL RNase (Thermo Scientific), and 1 µl/mL protease inhibitor cocktail (Sigma P8849)]. The cells were lysed by ultrasonication using a Braun-Sonic U ultrasonic cell disruptor on high setting, followed by centrifugation at 22,000 *g* at 4°C for 1.5 h. The recombinant His_6_-tagged Rho proteins were purified by nickel-chelation affinity chromatography using a Ni^2+^-nitrilotriacetic acid-agarose column (Qiagen, Valencia, CA), followed by tandem ion-exchange chromatography using HiTrap ANX-SP tandem columns (GE Healthcare), each eluted separately by using a salt gradient. The resulting purified Rho proteins were desalted by gel-filtration chromatography using a PD-10 column (GE Healthcare), eluting with PBS containing 10% glycerol. Each purified Rho protein was then subjected to MALDI mass spectral analysis using a Bruker Autoflex Speed LRF MALDI mass spectrometer (University of Illinois Urbana-Champaign Mass Spectrometry Laboratory).

### Cell culture

Human embryonic kidney epithelial HEK293T cells (ATCC #CRL-3216) were cultured in Dulbecco’s modified Eagle medium (DMEM; Gibco-Invitrogen, Grand Island, NY, USA), supplemented with 0.37% sodium bicarbonate, 100 U/mL penicillin-streptomycin (ThermoFisher Scientific), and 10% FetalPlex Animal Serum (AS; Gemini Bio-Products). Cells were maintained in DMEM with 5% AS and then switched to 2% AS at the time of transfection before experiments were performed.

### SRE-luciferase reporter gene assay for SRE activation

HEK293T cells in 10 mm culture dishes at 80% confluency were transfected using the previously described calcium phosphate method (75). Cells were cotransfected with a plasmid containing an SRE promoter fused to a firefly luciferase reporter gene (pSRE-luc, Stratagene) and another plasmid containing an HSV-TK promoter fused to a *Renilla* luciferase gene, acting as a low-expression constitutive reporter control gene (pGL4.74 hRluc/TK, Promega Madison, WI, USA) at a final DNA concentration of 1.6 µL/mL pSRE-luc and 0.2 µL/mL pGL4.74 hRluc/TK, respectively. Cells were incubated for 8 h at 37˚C, then replated into 48-well plates with fresh DMEM + 2% AS and incubated overnight (16–18 h). Toxin diluted in DMEM was then added to the wells containing transfected cells to give the final indicated concentration of the toxin. For acidification inhibitor experiments, fresh DMEM containing NH_4_Cl was added to the wells to give the indicated final concentrations and further incubated for 30 min before toxin treatment. After incubation for 6 h at 37˚C, cells were lysed using 50 µL of 1× Passive Lysis Buffer (Promega, Madison, WI, USA) per well for 15 min. Sample (10 µL) from each well was then transferred to a 96-well plate, and the lysates were analyzed for firefly luciferase reporter activity and the constitutive *Renilla* luciferase control activity, as previously described (28, 29). Reporter activity was measured using the Promega Dual-Luciferase Reporter 1000 Assay System by the addition of 25 µL of Luciferase Assay Reagent, followed by 25 µL of Stop and Glo Buffer per well, according to the manufacturer’s protocol. Experiments were performed at least three independent times or as indicated. For each experiment, all data points were performed in triplicate.

### RhoA gel shift assay of mammalian cell lysates

HEK293T cells were transfected with 10 µg/mL of pcDNA3.1-RhoA plasmid using the same procedure as the SRE reporter assay above. After an 8 h transfection period, cells were washed with 1× PBS and split into 6-well cell culture plates, and incubated at 37°C overnight. Toxin diluted in DMEM was then added to the wells containing transfected cells to give the final indicated concentration of the toxin. After incubation for 6 h at 37 ˚C, cells were lysed using 500 µL of 1× Passive Lysis Buffer (Promega, Madison, WI, USA) per well for 15 min. The cell lysates were transferred to 1.5 mL microcentrifuge tubes and then microcentrifuged at 13,000 rpm for 5 min, and the supernatant was transferred to new 1.5 mL tubes. To each tube, 100 µL of 5× SDS sample buffer was added, and samples were heated in boiling water for 10 min, followed by the addition of 100 µL of bromothymol blue dye. Then, 20 µL of each sample was subjected to SDS-PAGE analysis using gels with a tiered lower stack of 12.5% (top) and 15% (bottom) acrylamide. The resulting gels were analyzed by western blot as described above for the coexpression assays.

### Statistical analysis

EC_50_ values were determined by nls curve_fit function from scipy using the equation [*d* + (*a* − *d*)]/{1 + *e*^[−b * (*x* − *c*)]}, where *d* is the minimum asymptote, *a* is the maximum asymptote, *b* is the slope, and *c* is the point of inflection (or EC_50_ value). The *P*-values between the dose-response curves of each toxin were determined by applying a *t*-test to the dose curves using drc v3.0-1 in R package. The dose-response curves were generated using the data points from all replicates. The *P*-values between the curves were determined by comparing dose-response curves using the average values of the replicates within each repeated experiment. The resulting *P-*values between the dose-response curves for each toxin are listed in Table S2.

## Data Availability

All data accession numbers for nucleotide and amino acid sequences and protein structures used in this study were obtained or generated from existing databases as indicated in the figures, figure legends, and supplemental material.

## SUPPLEMENTAL MATERIAL

Supplemental Information mbio.01221-24-s0001.pdf Figures S1-S9 and Table S2.

Table S1 mbio.01221-24-s0002.xlsx CNFx genome metadata.

ASM does not own the copyrights to Supplemental Material that may be linked to, or accessed through, an article. The authors have granted ASM a non-exclusive, world-wide license to publish the Supplemental Material files. Please contact the corresponding author directly for reuse.
