# Supplementary material for: Hierarchical determinants in cytotoxic necrotizing factor (CNF) toxins driving Rho G-protein deamidation versus transglutamination
Source: mBio. 2024 Jun 26;15(7):e01221-24. doi: 10.1128/mbio.01221-24 (PMC11253639; doi:10.1128/mbio.01221-24)
Supplement: Supplemental Information — Figures S1-S9 and Table S2. [file mbio.01221-24-s0001.pdf]

Hierarchical determinants in cytotoxic necrotizing factor (CNF) toxins driving Rho G-protein deamidation versus transglutamination

Nicholas B. Handy,[a] Yiting Xu,[a]§ Damee Moon,[a]¶ Jacob J. Sowizral,[a] Eric Moon,[a] Mengfei Ho,[a] and Brenda A. Wilson[a]#

[a]Department of Microbiology, School of Molecular and Cellular Biology, University of Illinois at Urbana-Champaign, Urbana, Illinois, USA

Running Head: Determinants of CNF deamidation vs. transglutamination

# Address correspondence to Brenda A. Wilson, wilson7@illinois.edu

§ Present address: Department of Molecular, Cellular and Developmental Biology, Yale University, New Haven, Connecticut, USA. Email: Yiting.Xu@yale.edu

¶ Present address: New York University Grossman School of Medicine, New York, NY, USA. Email: Damee.Moon@nyulangone.org

SUPPLEMENTAL FIGURES

Figure S1. The CNFx-encoding gene resides on a putative 153.5-kb plasmid. A. Artemis Synteny Plot comparing the four largest *cnfx* gene-containing contigs (PTQN01000011.1, BFFX01000029.1, AATKTJ010000010.1, and JAAULO010000015.1) revealed overlapping sequences consistent with a *cnfx* gene (blue arrows) residing on a plasmid. **B.** BRIG output image of 15 selected *cnfx* gene-containing contig assemblies. Innermost ring: GC content (black). Second innermost ring: purple = GC skew (-), green = GC skew (+). Third innermost ring: The sequence of the circularized contig (PTQN01000011.1) was used as the reference sequence. Remaining solid-colored rings: The name of the genome assemblies for the contigs and their corresponding color codes are indicated in the upper right legend. Outermost ring: Highlighted are open reading frames with annotation. Black = *cnfx* gene, Red = Plasmid-specific genes, Green = Virulence genes, Blue = Other genes. A table listing all the open reading frames with annotation is provided in **Supplemental Table S1**.

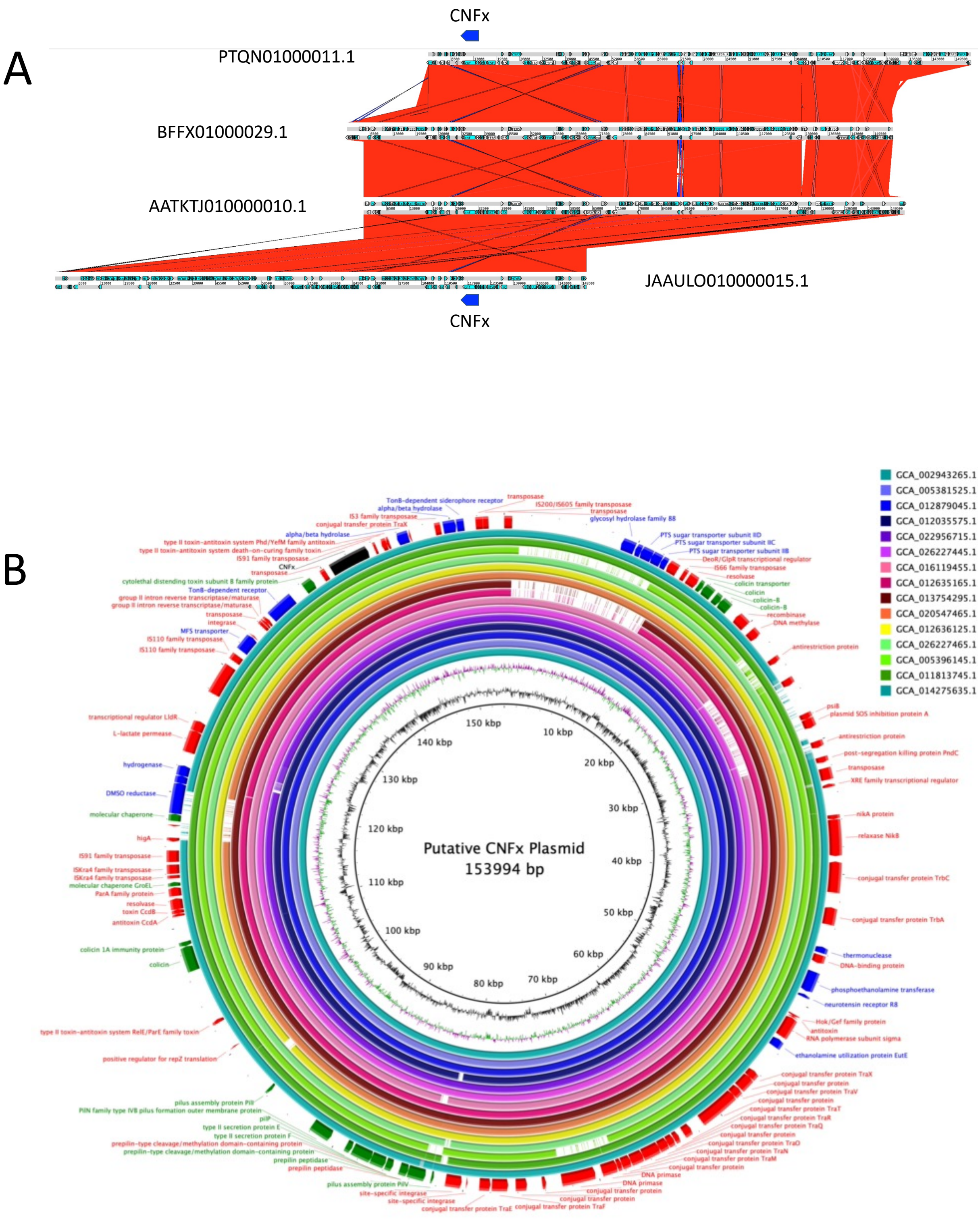
A
CNFx
PTQN01000011.1
BFFX01000029.1
AATKTJ010000010.1
JAAULO010000015.1
CNFx
B
GCA_002943265.1
GCA_005381525.1
GCA_012879045.1
GCA_012035575.1
GCA_022956715.1
GCA_026227445.1
GCA_016119455.1
GCA_012635165.1
GCA_013754295.1
GCA_020547465.1
GCA_012636125.1
GCA_026227465.1
GCA_005396145.1
GCA_011813745.1
GCA_014275635.1
Putative CNFx Plasmid
153994 bp
10 kbp
20 kbp
30 kbp
40 kbp
50 kbp
60 kbp
70 kbp
80 kbp
90 kbp
100 kbp
110 kbp
120 kbp
130 kbp
140 kbp
150 kbp
TonB-dependent siderophore receptor
alpha/beta hydrolase
IS3 family transposase
conjugal transfer protein TraX
transposase
IS200/IS605 family transposase
glycosyl hydrolase family 88
type II toxin-antitoxin system Phd/YefM family antitoxin
type II toxin-antitoxin system death-on-curing family toxin
IS91 family transposase
CNFx
cytolethal distending toxin subunit B family protein
TonB-dependent receptor
group II intron reverse transcriptase/maturase
integrase
MFS transporter
IS110 family transposase
PTS sugar transporter subunit IID
PTS sugar transporter subunit IIC
PTS sugar transporter subunit IIB
DeoR/GlpR transcriptional regulator
IS66 family transposase
resolvase
colicin transporter
colicin
colicin-B
recombinase
DNA methylase
antirestriction protein
psiB
plasmid SOS inhibition protein A
post-segregation killing protein PndC
XRE family transcriptional regulator
nikA protein
relaxase NikB
conjugal transfer protein TrbC
conjugal transfer protein TrbA
thermonuclease
DNA-binding protein
phosphoethanolamine transferase
neurotensin receptor R8
Hok/Gef family protein
antitoxin
RNA polymerase subunit sigma
ethanolamine utilization protein EutE
transcriptional regulator LldR
L-lactate permease
hydrogenase
DMSO reductase
molecular chaperone
higA
ISKra4 family transposase
molecular chaperone GroEL
ParA family protein
toxin CcdB
antitoxin CcdA
colicin 1A immunity protein
type II toxin-antitoxin system RelE/ParE family toxin
positive regulator for repZ translation
pilus assembly protein PilI
PilN family type IVB pilus formation outer membrane protein
pilP
type II secretion protein E
type II secretion protein F
prepilin-type cleavage/methylation domain-containing protein
prepilin peptidase
pilus assembly protein PilV
site-specific integrase
conjugal transfer protein TraE
conjugal transfer protein TraF
conjugal transfer protein
DNA primase
conjugal transfer protein TraV
conjugal transfer protein TraT
conjugal transfer protein TraR
conjugal transfer protein TraQ
conjugal transfer protein TraO
conjugal transfer protein TraN
conjugal transfer protein TraM

Figure S2. Purity and cellular activity of full-length wildtype toxins (CNF1, CNF3, CNFy, CNFx) and chimeric toxins (CNFxy, CNFx3, CNFyx). A. Coomassie-stained SDS-PAGE gels of purified proteins of wildtype CNF1, CNF3, CNFy, and CNFx toxins and chimeric toxins CNFx3, CNFxy, and CNFyx using the 519 joining site and CNFyx using the 688 joining site. **B.** Scatter plot for dose response curves for CNF-treated HEK293T cells. Cells transfected with reporter plasmids were treated with the indicated toxin at the indicated concentration for 6 h and then lysed and analyzed by SRE-luciferase assay, as described in Materials and Methods. Data points are from individual treatments for that specified dose for 3 independent repeats performed in triplicate.

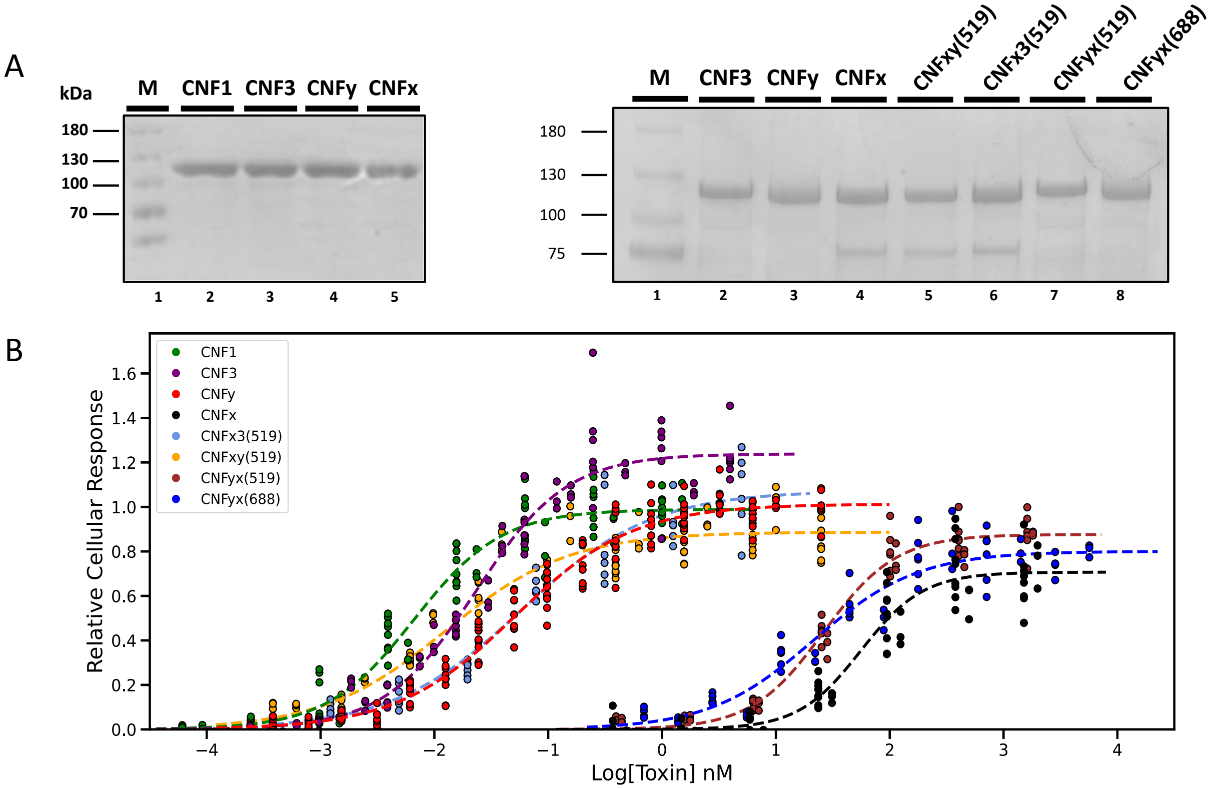

Figure S3. Modification of Rho proteins by CNF1 and CNFx. A. Additional repeats of gel-shift assay western blots of cell lysate from HEK293T cells treated with CNF1 at the indicated concentrations for 6 h, as described in Materials and Methods. **B.** Additional repeats of gel-shift assay western blots of cell lysate from HEK293T cells treated with CNFx at the indicated concentrations for 6 h, as describe in Materials and Methods. **C.** Scatter plot used for quantification of the RhoA gel shifts and determination of the EC_{50} values for CNF1 and CNFx. RhoA shift gels were analyzed using ImageJ to calculate the ratio of modified to unmodified RhoA and plotted using Python in the lower panel. CNF1 EC_{50} value = 0.79 nM, CNFx EC_{50} value = 125 nM.

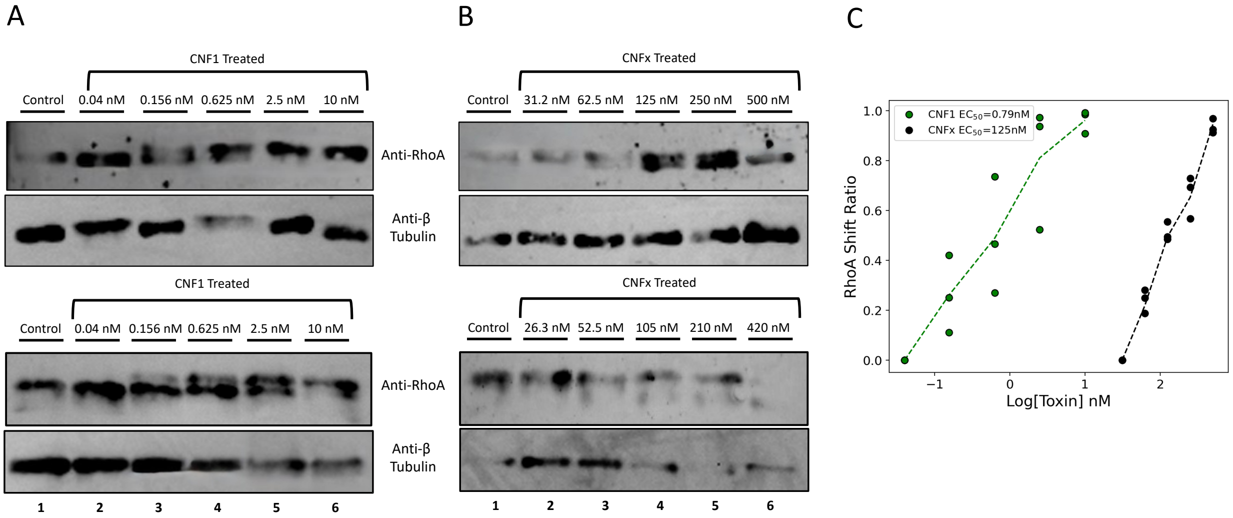

Figure S4. Purity and cellular activity of full-length wildtype and point mutants of CNF1 and CNFx. A. Coomassie-stained SDS-PAGE gel of purified wildtype CNF1 and CNFx and point mutants CNF1 (R832H), CNF1 (R832N), CNF1 (R832H, N862E), CNF1 (R832N, N862E), and CNFx (E857N) proteins. **B.** Scatter plot of SRE-luciferase dose response curves for wildtype and point mutants of the indicated CNF1 and CNFx. HEK293T cells with reporter plasmids were treated with the indicated toxin concentration for 6 h and then lysed and analyzed by SRE-luciferase assay, as described in Materials and Methods. Data points for individual treatments are for that specified dose for 3 independent repeats performed in triplicate.

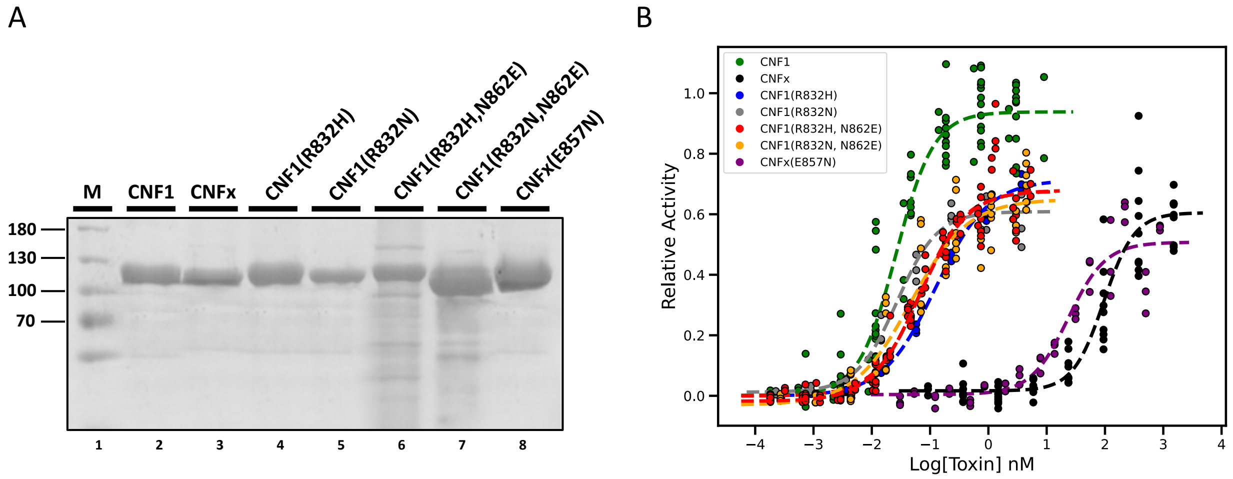

Figure S5. Structural models of the C-terminal catalytic domains of the CNF toxins. For each of the toxins (CNF1, CNF2, CNF3, CNFy, CNFx, and CNFm1), sequences from the start of the catalytic domains (corresponding to position 735 in CNF1) were used to generate structural models in Modeller **(A)** or Swiss-Modeller **(B)** using the known crystal structures containing CNFy and CNF1 C-terminal domain (6YHK, 6YHM, 6YHN, and 1HQ0 pdb), as described in the Materials and Methods. Visualizations were performed in ChimeraX. In the ribbon models, the mutated residues are indicated. In all of the models, the catalytic cysteine is shown in yellow, and the catalytic histidine is shown in cyan.

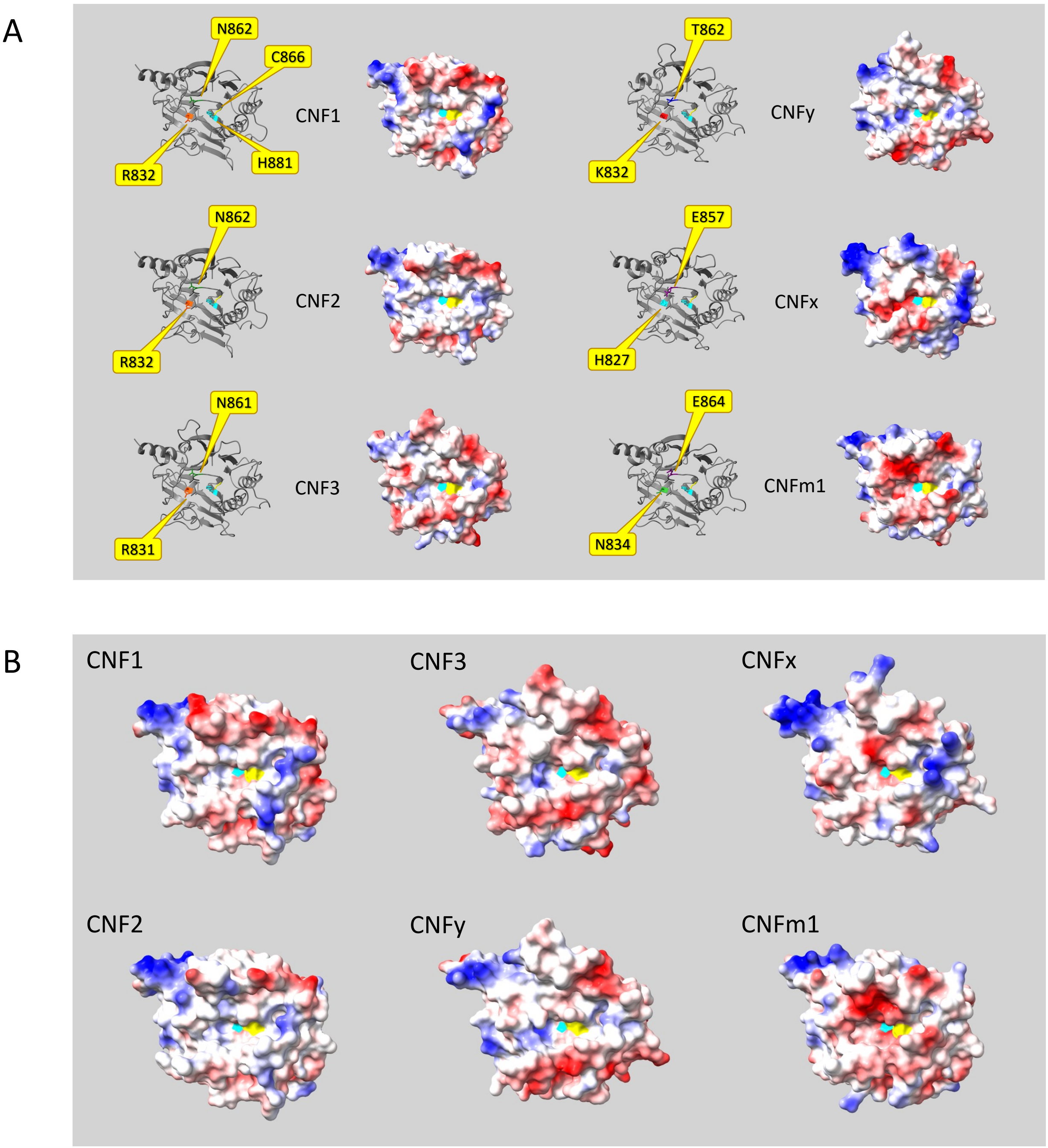
A
N862
C866
CNF1
R832
H881
T862
CNFy
K832
N862
CNF2
R832
E857
CNFx
H827
N861
CNF3
R831
E864
CNFm1
N834
B
CNF1
CNF3
CNFx
CNF2
CNFy
CNFm1

Supplemental Figure S6. Amino acid sequence alignment of CNF toxin homologs. A. The protein sequence alignment was generated in COBALT, using sequences obtained from NCBI: CNF1 Accession #WP_000528124.1, CNF2 Accession #WP_033559220.1, CNF3 Accession #WP_024231387.1, CNF4 Accession #EFM9255861.1, CNF5 Accession #MBB7655459.1, CNFcm Accession #PLS41055.1, CNFm1 Accession #WP_045110427.1, CNCm2 Accession #WP_075533205.1, CNFp Accession #WP_005306733.1, CNFse Accession #WP_079952502.1, CNFy Accession #WP_012304286.1, CNFyr Accession #EKN4209683.1, CNFx Accession #WP_059330985.1, and DNT Accession #WP_010931478.1. **B.** Repeats of RhoA gel-shift assays for expression of RhoA alone or coexpression with CNF1, DNT, or DNT (S1304R) in E. coli BL21 cells. Top 2 panels: Coomassie-stained SDS-PAGE gels; Bottom panel: Western blot using anti-HA antibodies.

A

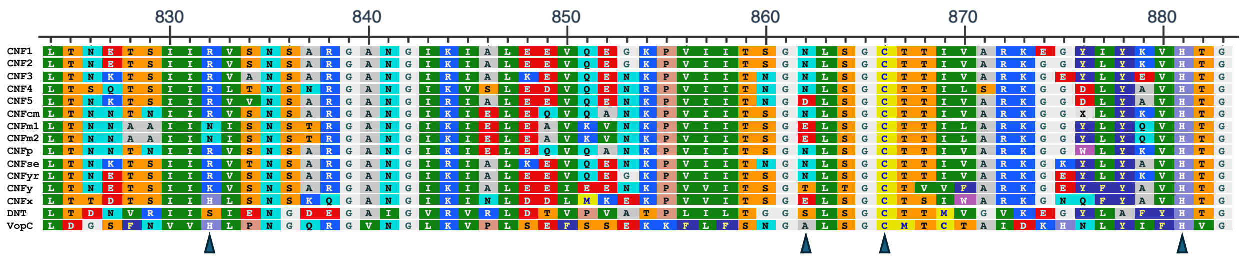

B

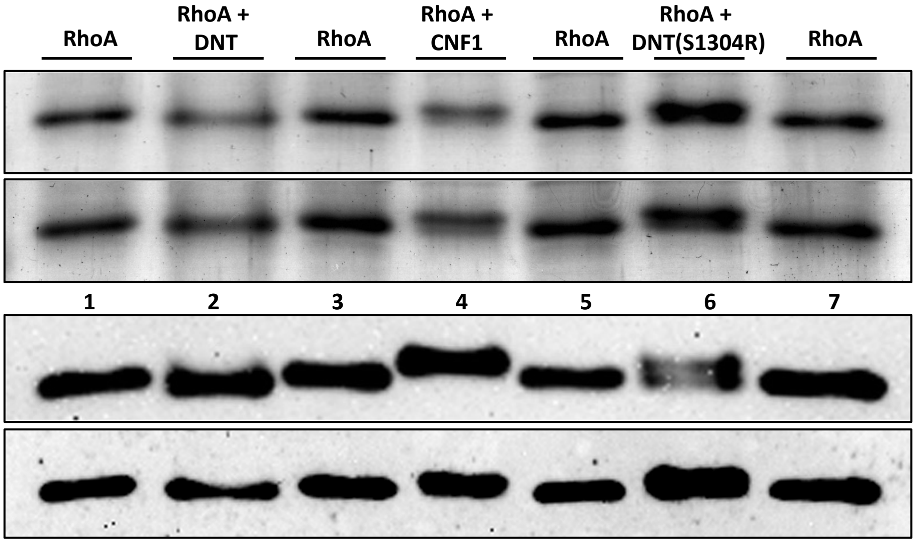

Figure S7. Time course of full-length wildtype and point mutants of CNF1 and CNFx. A. Time course of SRE cellular response activity for each of the indicated toxins. HEK293T cells with reporter plasmids were treated with the indicated toxin for 6 h and then lysed and analyzed by SRE-luciferase assay, as described in Materials and Methods. Data points shown are the mean values for that specified dose from at least 3 independent repeats performed in triplicate. **B.** Shown is the corresponding scatter plot with all data points used to derive the best fit lines and mean values for (A).

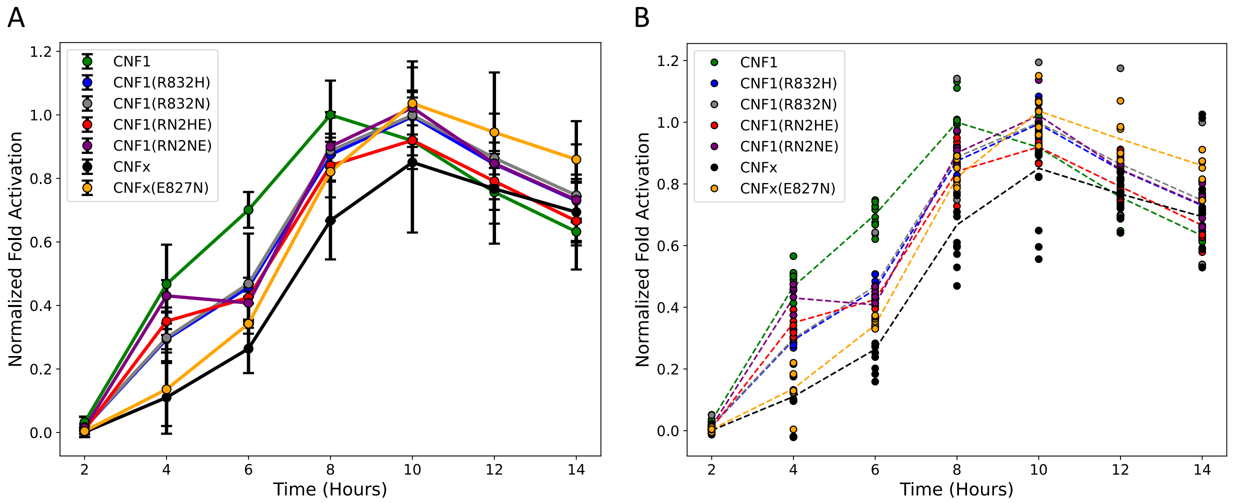

Figure S8. Purity and cellular activity of full-length wildtype and Cys mutants of CNF1 and CNFx. A. Coomassie-stained SDS-PAGE gel of purified wildtype CNF1 and CNFx proteins and point mutants CNFx (E857N, C1005S) and CNFx (C1005S). **B.** Scatter plot of dose response curves for CNFx chimera toxin treated HEK293T cells for **Figure 7B**. HEK293T cells with reporter plasmids were treated with the indicated toxin concentration for 6 h and then lysed and analyzed by SRE-luciferase assay, as described in the Materials and Methods. Data points shown are the mean values for that specified dose from 3 independent repeats performed in triplicate. **C.** Additional repeats of gel-sift assay western blots of cell lysates from HEK293T cells treated with CNFx (C1005S) at the indicated concentrations for 6 h, as describe in Materials and Methods. **D.** Additional repeats of gel-shift assay western blots of cell lysates from HEK293T cells treated with CNFx (E857N, C1005S) at the indicated concentrations for 6 h, as describe in Materials and Methods. **E.** Scatter plot used for quantification of the RhoA gel shifts and determination of the EC_{50} values for CNFx (C1005S) and CNFx (E857N, C1005S). RhoA shift gels were analyzed using ImageJ to calculate ratio of modified to unmodified RhoA and plotted using Python. **F.** Shown is the corresponding scatter plot with all data points used to derive the best fit lines and mean values for **Figure 7D**.

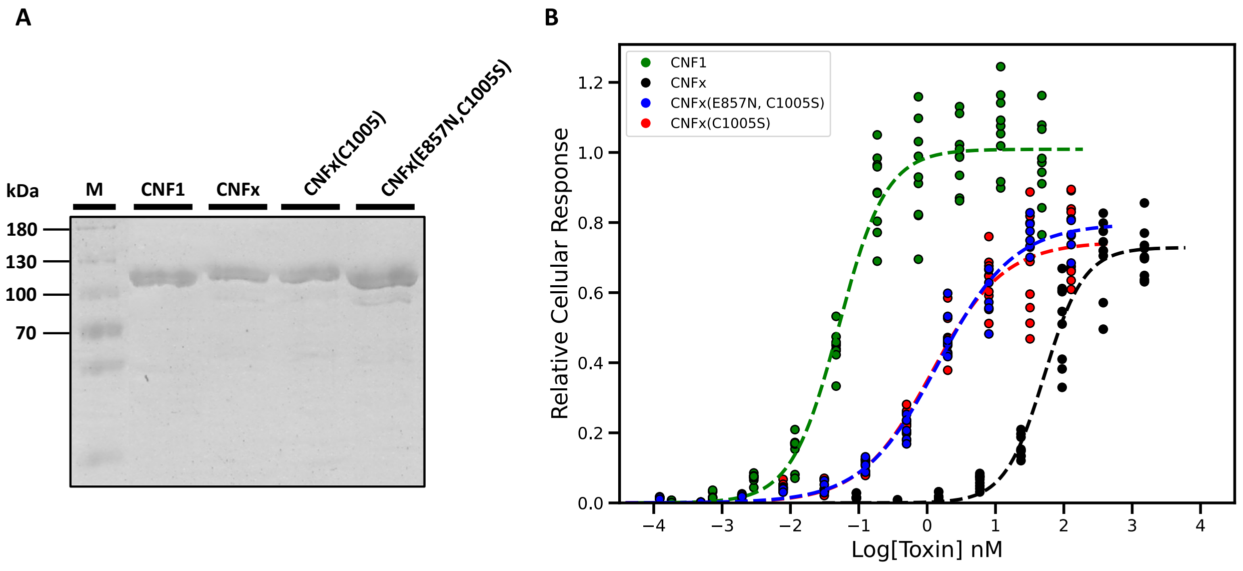

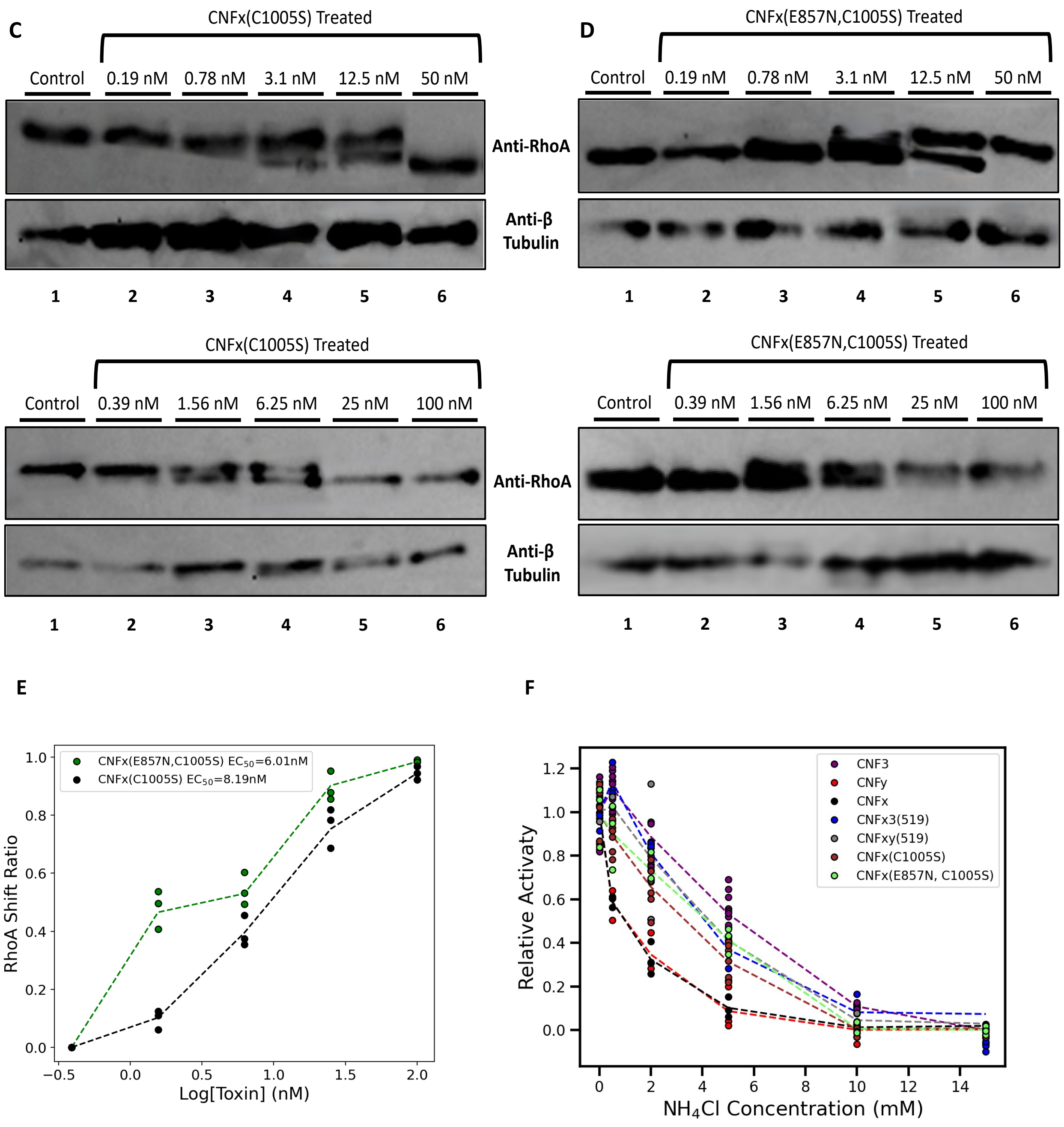
C
CNFx(C1005S) Treated
Control
0.19 nM
0.78 nM
3.1 nM
12.5 nM
50 nM
Anti-RhoA
Anti-β Tubulin
1 2 3 4 5 6
CNFx(C1005S) Treated
Control
0.39 nM
1.56 nM
6.25 nM
25 nM
100 nM
Anti-RhoA
Anti-β Tubulin
1 2 3 4 5 6
D
CNFx(E857N,C1005S) Treated
Control
0.19 nM
0.78 nM
3.1 nM
12.5 nM
50 nM
1 2 3 4 5 6
CNFx(E857N,C1005S) Treated
Control
0.39 nM
1.56 nM
6.25 nM
25 nM
100 nM
1 2 3 4 5 6
E
CNFx(E857N,C1005S) EC50=6.01nM
CNFx(C1005S) EC50=8.19nM
RhoA Shift Ratio
Log[Toxin] (nM)
F
CNF3
CNFy
CNFx
CNFx3(519)
CNFxy(519)
CNFx(C1005S)
CNFx(E857N, C1005S)
Relative Activaty
NH4Cl Concentration (mM)

Figure S9. Phylogenetic relationship of CNF C-terminal homologs.

Shown is a phylogenetic tree of 163 representative sequences homologous to the CNF1 C-terminus, with accession numbers and source bacteria indicated, generated using ggtree in R.

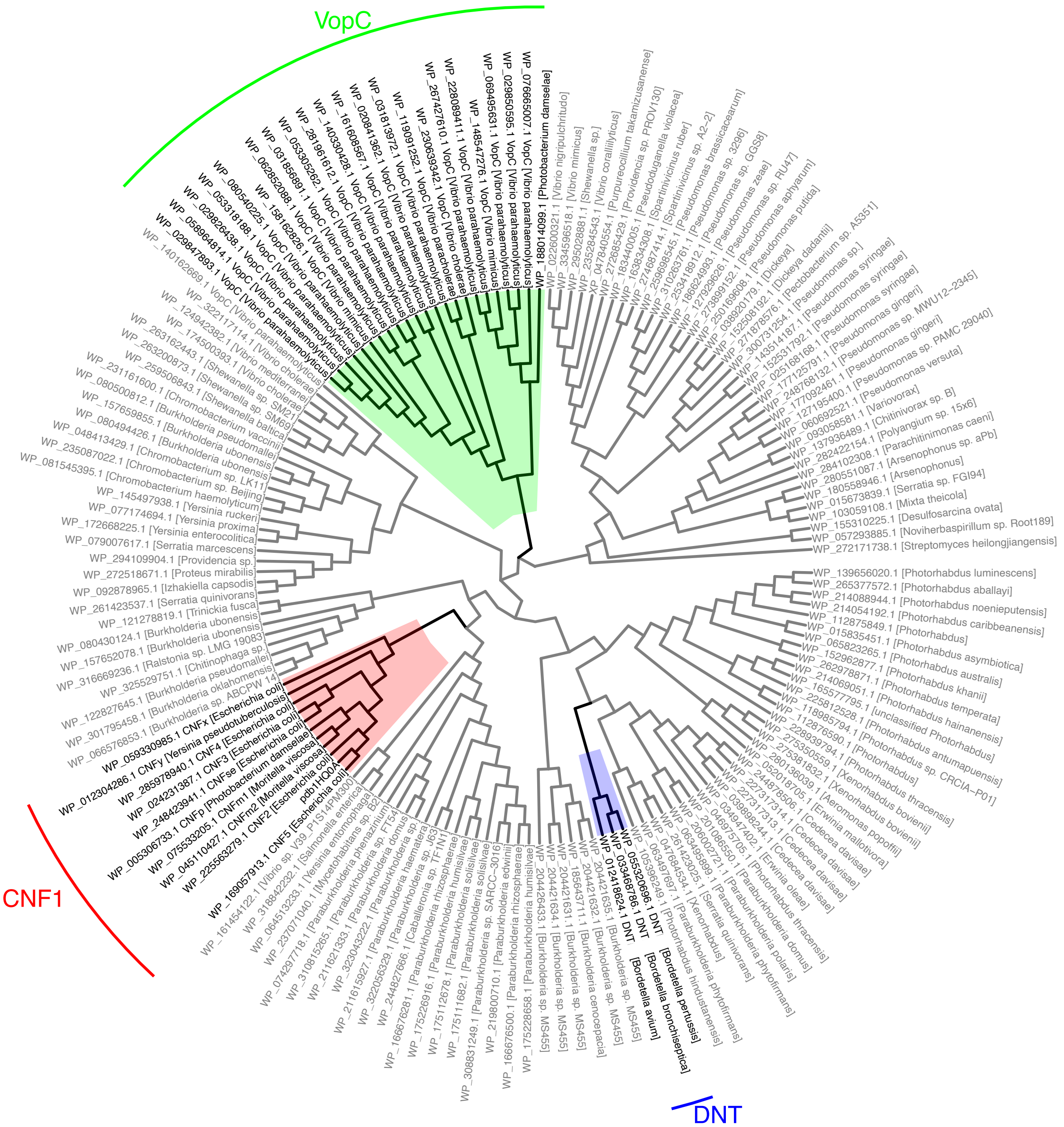

SUPPLEMENTAL TABLES

Supplemental Table S1. List of all open reading frames in putative *cnfx* gene-containing 153.5-kb plasmid.

CNFx_genome_metadata.xlsx

Supplemental Table S2. *P-values* for toxin dose-response curves.

A

	CNF1	CNF3	CNFy	CNFx	CNFxy(519)	CNFx3(519)	CNFyx(519)
CNF1							
CNF3	5.02E-09						
CNFy	3.54E-04	2.39E-02					
CNFx	1.50E-05	1.50E-05	1.52E-05				
CNFxy(519)	3.60E-02	2.60E-02	3.38E-03	1.50E-05			
CNFx3(519)	4.98E-04	1.40E-02	5.86E-01	1.52E-05	2.77E-03		
CNFyx(519)	4.40E-11	4.50E-11	4.68E-11	3.48E-02	4.44E-11	4.76E-11	
CNFyx(688)	9.18E-05	9.28E-05	9.45E-05	2.47E-02	9.22E-05	9.52E-05	6.19E-01

B

	CNF1	CNFx	CNF1(R832H)	CNF1(R832N)	CNF1(R832H, N862E)	CNF1(R832N, N862E)
CNF1						
CNFx	1.50E-05					
CNF1(R832H)	2.45E-02	2.86E-13				
CNF1(R832N)	9.16E-01	2.72E-13	2.79E-02			
CNF1(R832H, N862E)	5.62E-04	2.78E-13	1.93E-01	4.35E-03		
CNF1(R832N, N862E)	3.61E-02	2.75E-13	8.32E-02	1.04E-01	2.06E-01	
CNFx(E857N)	9.28E-06	2.11E-07	1.01E-05	9.29E-06	9.60E-06	9.45E-06

C

	CNF1	CNFx	CNFx(E857N, C1005S)
CNF1			
CNFx	1.50E-05		
CNFx(C1005S)	1.26E-10	7.61E-22	
CNFx(E857N, C1005S)	3.31E-07	1.26E-21	3.74E-01

p-val < 0.001
p-val < 0.01
p-val < 0.05
p-val > 0.05
